# Highly *E*‑Selective Alkene Isomerization Using Me_4_NF at Room Temperature

**DOI:** 10.1021/acs.joc.5c02159

**Published:** 2025-11-24

**Authors:** Leah Webster, Rofiat Ayoola Shoetan, Catherine M. Alder, Ruth L. Webster

**Affiliations:** † Yusuf Hamied Department of Chemistry, 2152 University of Cambridge, Lensfield Road, Cambridge CB2 1EW, U.K.; ‡ Department of Chemistry, 1555 University of Bath, Claverton Down, Bath BA2 7AY, U.K.; § GSK, Gunnels Wood Road, Stevenage SG1 2NY, U.K.

## Abstract

Allylbenzene isomerization to trans-β-methylstyrene is catalyzed by Me_4_NF (40 mol %) in dry CD_3_CN under an inert atmosphere at room temperature, delivering high E-selectivity (*E*:*Z* = 100:0). The method tolerates diverse substituents but is limited by polar, protic groups. Some preliminary mechanistic studies are also reported.

## Graphical abstract

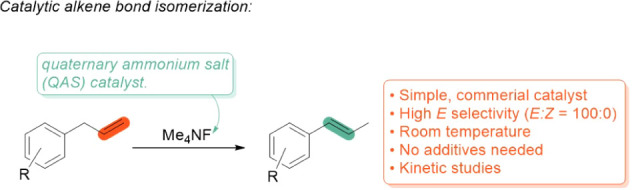

## Introduction

Carbon–carbon double bonds are an important functional group in synthetic chemistry; they can be functionalized using a range of different methodologies, along with being cleaved, homologated, or isomerized. However, the latter can often be a bottleneck in the multistep synthesis of organic molecules. The importance of double bond isomerization means that a huge range of methods have been developed to successfully afford this transformation.^1,2^ This includes acid- and base-mediated methods,^3^ along with transition metal- and main group-catalyzed processes that employ anything from a simple metal salt—some of the most common being palladium^4–7^ and rhodium^8–12^ —through to carefully designed ligated systems, and more recently, hydrogen atom transfer catalysis and visible light-mediated systems.^13–17^ An area of focus for isomerization reactions has been that of allyl-to-vinyl reactions, especially those based on the propenyl-benzene fragment. These fragments are often found in nature, and isomerization to the β-methylstyrene is important for transformations at a different, more desirable position, often being a key step in ring-closing metathesis and in forming bioactive molecules.^18–20^ Although driven by the thermodynamic payback of generating a conjugated styrenyl product, challenges still remain in this seemingly simple bond transposition. For example, base-catalyzed reactions rarely show complete selectivity for the *E* or *Z* isomer, and often require excess base and high temperatures for isomerization to occur. Recently, this research area has progressed, with nontransition-metal catalysts now showing good conversion and higher *E*/*Z* selectivity, alongside milder conditions and lower catalytic loadings.^13,16,21,22^ For example, Hevia and coworkers^21^ have utilized a sodium amide and Lewis-donor combination for the isomerization of allyltrimethylsilane, with a 10 mol % catalyst loading at room temperature, yielding the internal alkene with yields of 72%, and a 94:6 *E:Z* ratio. There have also been some elegant advances made using transition metal catalysts to access the challenging contra-thermodynamic Z-isomer^23–26^ and for complete *E* selectivity.^27^ For example, the work of Holland and coworkers^24^ showed a *Z:E* ratio for the isomerization of allylbenzene of 93:7 with a cobalt­(I) β-dialdiminate catalyst.

Given the simplicity of the transformation, in that it is a bond transposition, we wondered whether there may be a simple way to effect this transformation without requiring an elaborate (and often air-sensitive) metal catalyst, while still showing good activity and selectivity for the *E*-isomer. We were intrigued by the work of Sasson and de la Zerda who studied tetra-*n*-butyl quaternary ammonium salts (QAS) in the isomerization of allylbenzene.^28–31^ These studies used isomerization primarily as a vehicle to understand decomposition pathways of QAS i.e., quaternization of the ammonium salt (reverse Menshutkin reaction)^32^ or Hoffman degradation of the ammonium salt,^28^ where one of the alkyl groups on the ammonium cation is lost as an alkene, leading to the formation of HX (where X is the ammonium counteranion) and the tertiary amine. Although the hydroxide counteranion is noted to lead to a higher rate of isomerization than an alkoxide counteranion, the accessible decomposition pathways inherently lead to a loss in catalysis. We therefore postulated that the use of nonphase-transfer conditions (as an example, a dry, nonprotic/base-free environment under inert conditions), and by avoiding long-chain alkyl ammonium reagents that can eliminate as an alkene, we might identify a highly active, but simple and commercially available isomerization catalyst (Scheme 1). Other examples where a phase-transfer QAS catalyst has been used in isomerization chemistry invariably employ a strong base (e.g., KOH, NaOH at 90–130 °C).^31–33^ Phenyltrimethylammonium methosulfate and K_2_CO_3_ in refluxing DMF has also been employed stoichiometrically,^33^ while tetramethylammonium chloride (1.25 equiv) has been employed in the presence of NaOH (1.25 equiv) in PEG at 150–160 °C.^34^ This latter study promoted the methylation of the free phenol of eugenol, along with isomerization. The *E*/*Z* ratio was 4:1. Although not optimized and instead reported as part of a study on cyclocondensation, ammonium acetate has been shown to facilitate double bond isomerization of allylbenzenes. Beyond this, QAS have been combined with transition metals for double bond isomerization^35,36^ at raised temperatures with moderate yields and ratios of the *E*/*Z* product.^37^

**Scheme 1:**
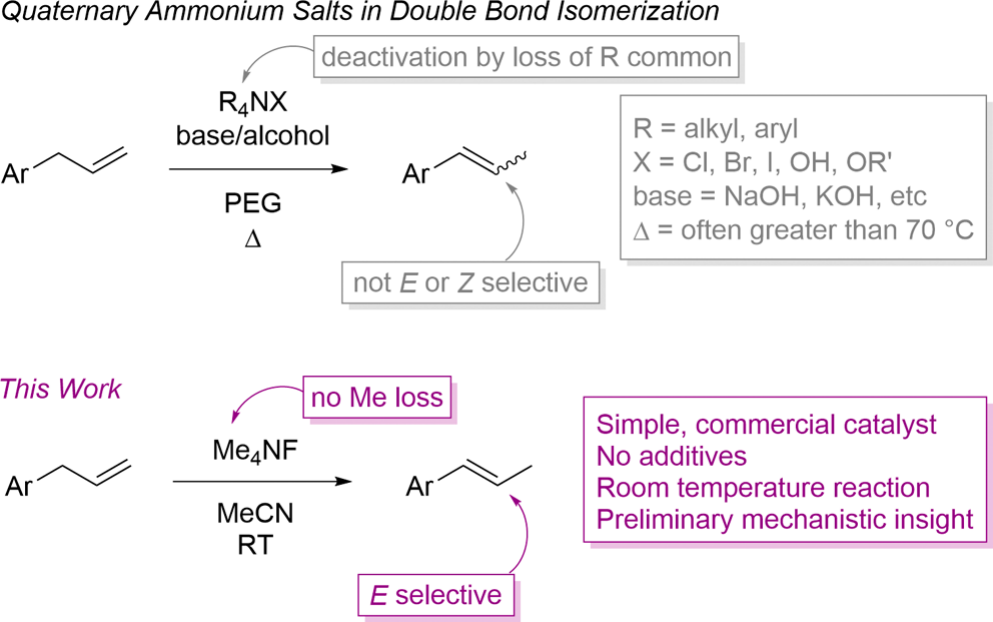
Quaternary Ammonium Salts in Double Bond Isomerization^a^

a Previous examples of double bond isomerization have employed quaternary ammonium salts, but often suffer from catalyst deactivation, poor stereoselectivity, and the need for forcing reaction conditions (top). In this work (bottom), tetramethylammonium fluoride is employed as a highly active and highly *E*-selective isomerization catalyst

The isomerization of allylbenzene was targeted for optimization purposes. ^n^Bu_4_NBr, Me_4_NBr, ^n^Bu_4_NF, and H_4_NF do not give any double bond isomerization product, but complete spectroscopic conversion of allylbenzene to *trans*-β-methylstyrene (**2a**, Scheme 2) was achieved using 40 mol % Me_4_NF at room temperature (RT). This indicates that it is both the quaternary ammonium cation and the presence of a fluoride that are important. Conversion drops below 10% when the Me_4_NF loading is less than 25 mol %; increasing the temperature to 80 °C and shaking for 2 h does not improve the yield. Reactions performed under an ambient atmosphere do not generate product, regardless of Me_4_NF loading. This is attributed to the hygroscopic nature of Me_4_NF, which likely forms Me_4_NOH, which requires base and high temperatures to afford the isomerized product.^29^ Given that Me_4_NF is commercially available and, although hygroscopic, easy to handle, 40 mol % Me_4_NF, dry CD_3_CN, and an inert atmosphere were employed to ensure maximum conversion of all substrates (Scheme 2).

**Scheme 2:**
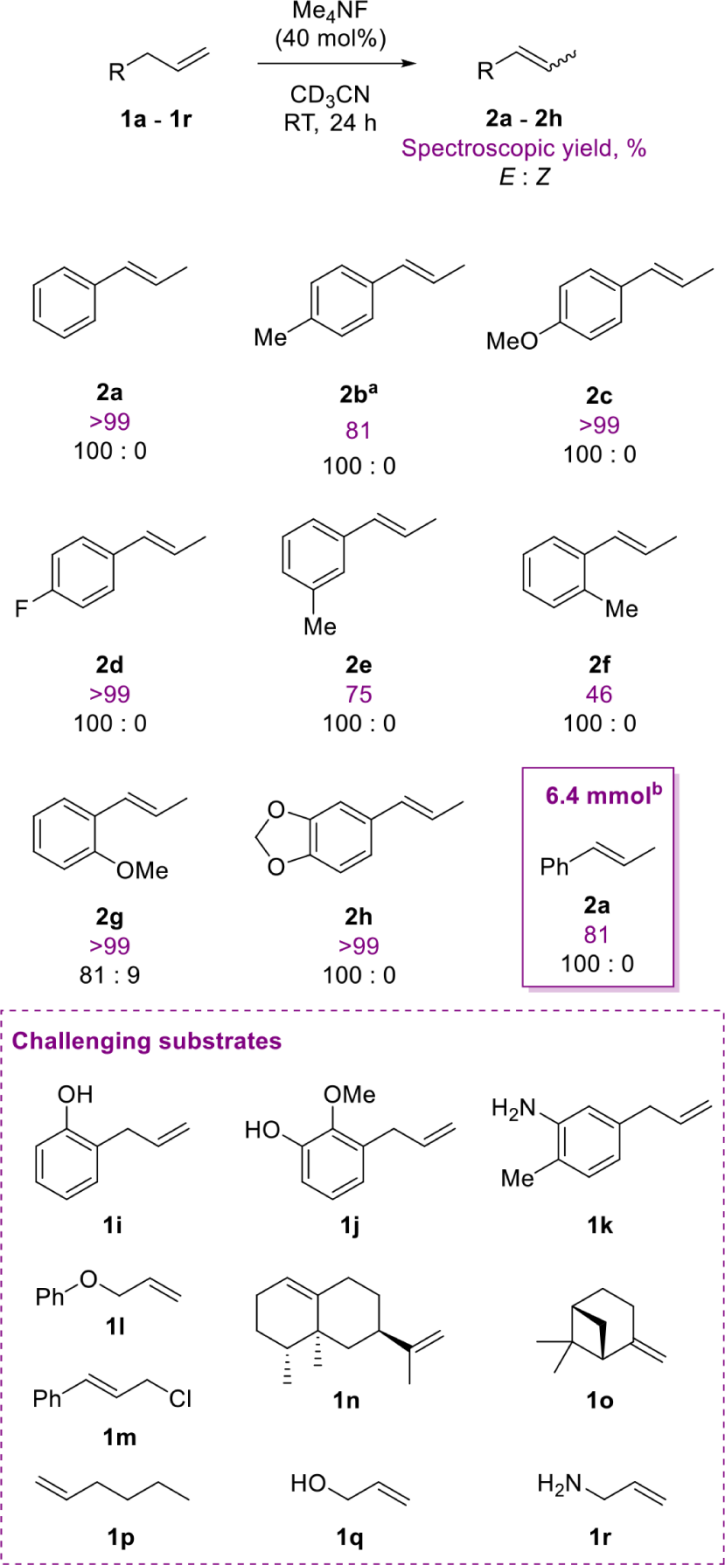
Substrate Scope, Including Disclosure of Challenging Substrates in Double Bond Isomerization Catalyzed by Me_4_NF at Room Temperature^a^

a Conversion obtained using 50 mol % Me_4_NF, which drops to 41% using 40 mol % Me_4_NF. ^b^40 mol % Me_4_NF, 27 h, RT

Extending the scope, a series of alkenes (**1a**–**1q**) were subjected to the transformation using Me_4_NF (40 mol %) as the catalyst in CD_3_CN at RT over 24 h. The transformation affords the corresponding styrenes (**2a**–**2h**) with excellent *E*-selectivity. Substrates bearing electron-donating (methoxy, methyl) or electron-withdrawing (fluoro) substituents on the aromatic ring are generally well tolerated. *Para*-substituted allyl arenes, such as *p*-methylallylbenzene (**1b**) and *p*-methoxyallylbenzene (**1c**), yield products **2b** and **2c** in 81% and quantitative yield, respectively, with exclusive formation of the *E*-isomer (*E:Z* = 100:0). Similarly, 4-fluoroallylbenzene (**1d**) provides product **2d** quantitatively with complete *E*-selectivity. *Ortho*- and *meta*-substituted allyl arenes also work well, although with somewhat diminished yields (**2e**, 75%; **2f**, 46%). Notably, substrates containing sterically crowded or fused aromatic systems, such as 2-methoxyallylbenzene (**1g**) and safrole (**1h**), delivered the corresponding products (**2g**, **2h**) in excellent yields (>99%). Although complete *E*-stereoselectivity is observed for **2h**, a small quantity of the *Z*-isomer is observed when **2g** forms. We attribute this to a stabilizing chelate effect from the -OMe group, which aids the *Z*-selective transition state. The reaction responds to scale-up, with 6.4 mmol (760 mg) of allylbenzene converting to **2a** in 81% spectroscopic yield after 27 h at RT. In contrast, several substrates do not undergo the desired transformation. These include substrates bearing free hydroxy groups (**1i**, **1j**), amino groups (**1k**, **1r**), benzylic ethers and halides (**1l**, **1m**), and aliphatic or nonaromatic olefins (**1p**, **1q**). Additionally, aliphatic, sterically encumbered systems such as valencene (**1n**) and β-pinene (**1o**) are also unreactive under the optimized conditions. The reaction outcome does not change for these substrates using forcing conditions (stoichiometric Me_4_NF, 80 °C, 24 h of agitation). The outcome with protic substrates could indicate that interaction with the fluoride catalyst is detrimental to the reaction outcome, i.e., counteranion exchange occurs, where a phenoxide anion is a less active QAS, in line with previous studies from Sasson, where strong base and heating are required for isomerization to take place.^31^ Increasing Me_4_NF to 1.5 equiv does not restore reactivity.

Mechanistically, we observe a pseudo-first-order reaction profile under our standard reaction conditions, indicating that although the loading of Me_4_NF is relatively high, it is acting as a catalyst. Me_4_NF is observed during catalysis, indicating that it may be on-cycle throughout the reaction. There is a short induction period, which we link to the time required for Me_4_NF to start to transfer into solution (see Supporting Information for *in situ* NMR data). We assume that the QAS first interacts closely with the allyl substrate, displacing fluoride and forming an allyl anion. The high *E*-selectivity of the reaction could be due to the steric bulk of the QAS, where long-range contact^38^ between the cation and anion promotes the *E-*isomer to form selectively at this stage. The sealed system means that HF released into solution is then able to reprotonate the allyl anion to deliver the most thermodynamically stable species (*E*-isomer), Scheme 3. F^–^ is known to deprotonate MeCN,^39^ and *in situ* ^19^F NMR monitoring shows that HF_2_^–^ forms during the course of the isomerization, as evidenced by a doublet in the ^19^F NMR spectrum (−145 ppm, ^1^ *J*_HF_ = 121 Hz). Commensurate with this result is the formation of DF_2_^–^ when CD_3_CN is employed; a 1:1:1 triplet at −147 ppm with ^1^ *J*_DF_ = 18 Hz is observed. Monitoring the reaction by ^19^F NMR spectroscopy, we observe a fairly rapid increase in the quantity of DF_2_^–^ in the reaction mixture (Chart [Fig chart1]), along with a comparable loss of Me_4_NF, followed by a slower decrease in the concentration of Me_4_NF in solution over the remainder of the reaction (Chart [Fig chart2]). No other fluorine-containing intermediates were observed during this study. To avoid this side reaction and test whether the Me_4_NF loading can be reduced, C_6_D_6_ and THF were tested as the reaction solvents. However, under the standard reaction conditions (40 mol % Me_4_NF), the reaction in C_6_D_6_ only gives 35% conversion to **2a**, but in a 94:6 ratio of the *E:Z* isomers, while THF gives 80% **2a** with complete selectivity for the *E* isomer. From this, we conclude that a polar solvent is necessary for high conversion (likely to aid solubility of Me_4_NF), but that formation of HF_2_^–^ is not detrimental to reactivity; indeed, generation of HF en route to this species may allow for better catalysis.

**Scheme 3:**
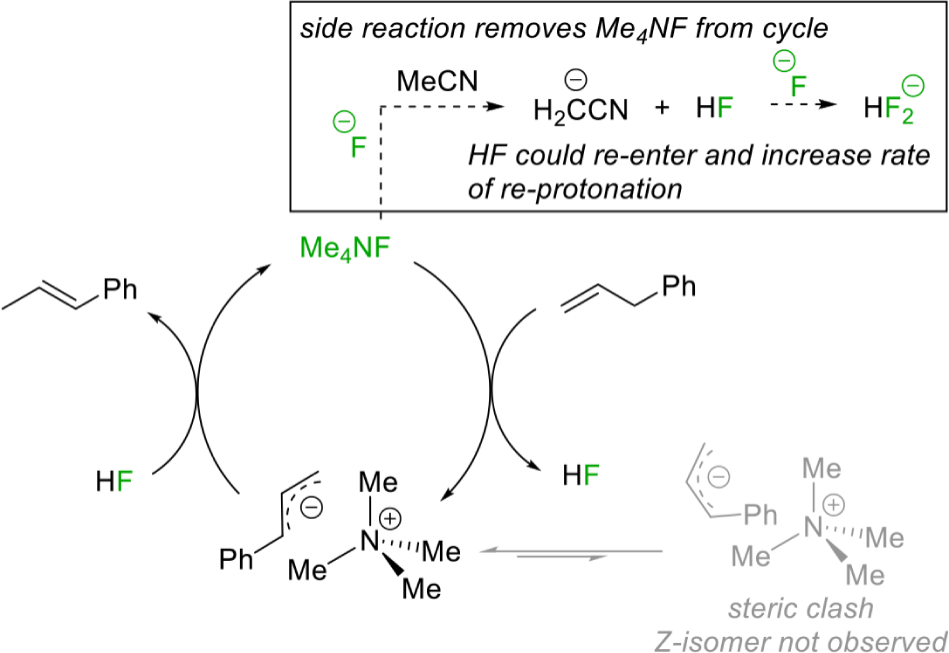
Postulated Catalytic Cycle

**1 chart1:**
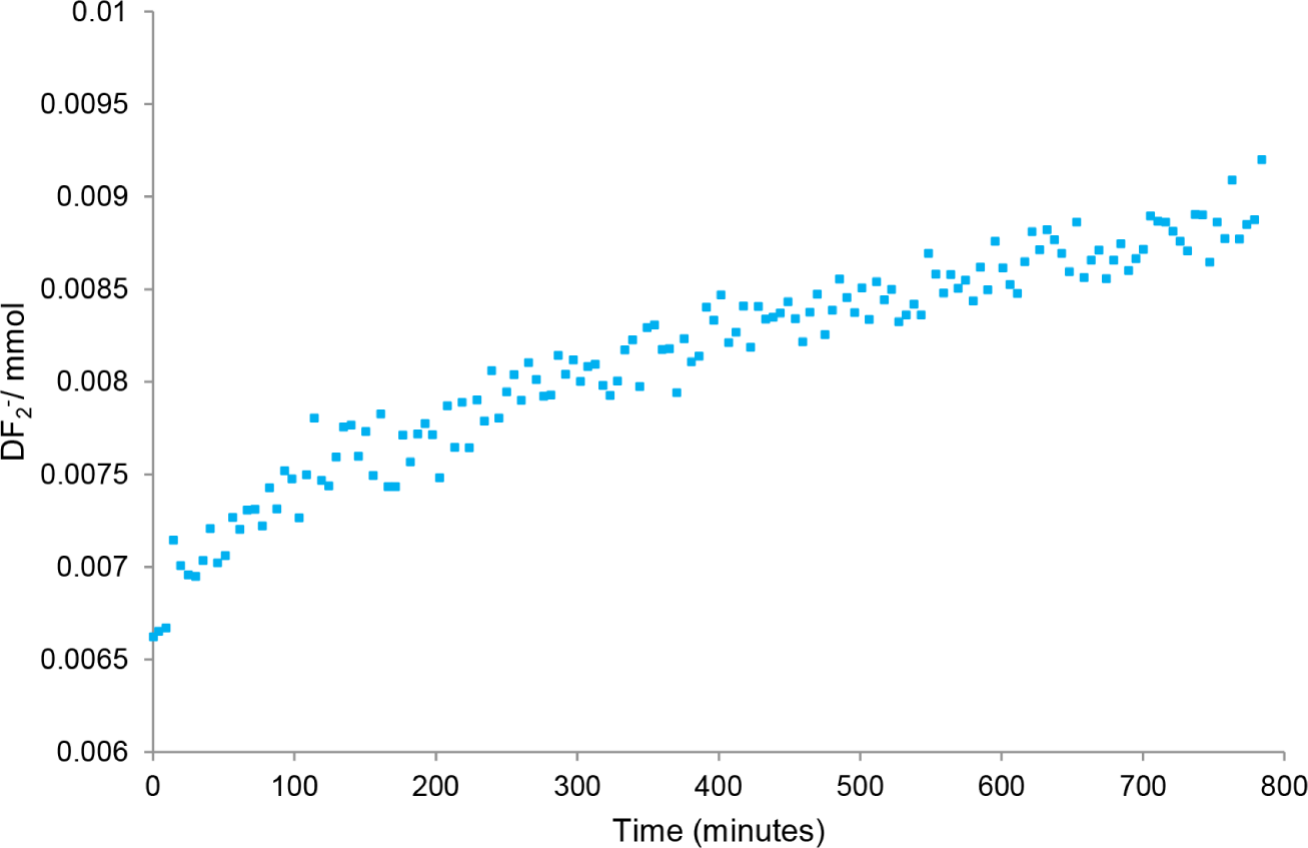
Moles of DF_2_^–^ as a Function of Time Collected Using ^19^F­{^1^H} NMR Spectroscopy^a^

a Reaction conditions: 0.25 mmol allylbenzene, 0.5 mL CD_3_CN, 40 mol %, room temperature

**2 chart2:**
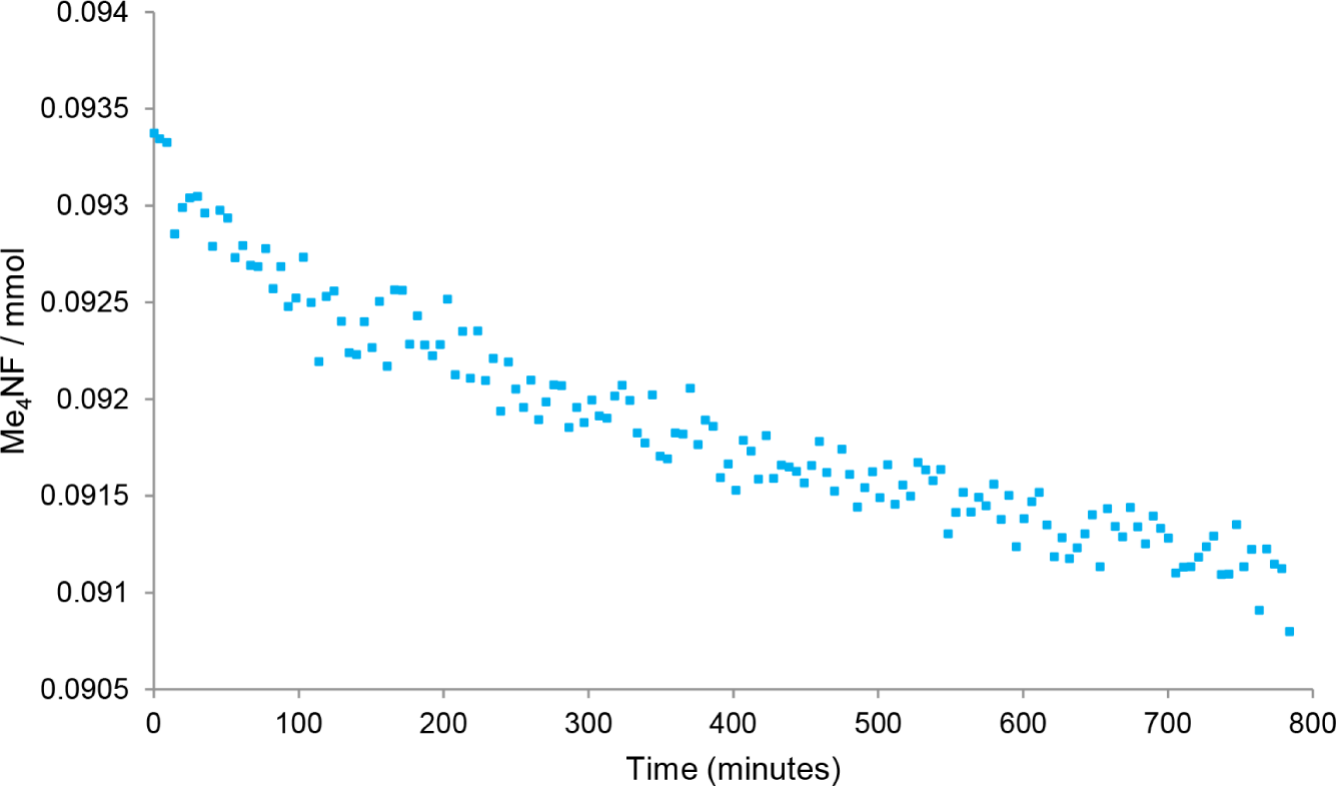
Moles of Me_4_NF as a Function of Time Collected using ^19^F­{^1^H} NMR Spectroscopy^a^

a Reaction conditions: 0.25 mmol allyl benzene, 0.5 mL CD_3_CN, 40 mol %, room temperature.

## Conclusion

To summarize, we have developed an operationally simple method to isomerize double bonds using a commercially available catalyst. This is vastly different from some of the elaborate transition-metal-catalyzed processes reported to date. The transformation displays broad functional group tolerance among aromatic substrates and delivers high stereoselectivity (*E:Z* = 100:0). However, the methodology is limited by poor compatibility with polar protic groups and nonaromatic or sterically hindered systems. To the best of our knowledge, we are not aware of any isomerization system that is so selective for the *E*-isomer and that operates under such simple and mild reaction conditions.

## Supplementary Material



## Data Availability

The data underlying this study are available in the published article, in its Supporting Information, and are openly available in the Open Science Framework repository, DOI: 10.17605/OSF.IO/3QTPY

## Notes

Additional experimental details, materials, methods, and NMR spectra are provided (PDF)
