# Supplementary material for: Highly E‑Selective Alkene Isomerization Using Me4NF at Room Temperature
Source: J Org Chem. 2025 Nov 24;90(48):17198–202. doi: 10.1021/acs.joc.5c02159 (PMC12690582; doi:10.1021/acs.joc.5c02159)
Supplement: Supplementary file 1 [file jo5c02159_si_001.pdf]

Supplementary Information

Highly *E*-Selective Alkene Isomerization Using Me_4NF at Room Temperature

Leah Webster,[a] Rofiat Ayoola Shoetan,[b] Catherine M. Alder,[c] Ruth L. Webster.[a]*

[a] *Yusuf Hamied Department of Chemistry, University of Cambridge, Lensfield Road, Cambridge, UK. CB2 1EW.*
[b] *Department of Chemistry, University of Bath, Claverton Down, Bath, UK. BA2 7AY.*
[c] *GSK, Gunnels Wood Road, Stevenage, UK. SG1 2NY*

Email: rw740@cam.ac.uk

1. Experimental Details 2
1.1 General Experimental Details 2
1.2 General Procedure 2
1.3 Scale up procedure of Allylbenzene, 2a. 2
1.4 Characterisation of products 2
2. Kinetic Data 5
2.1 General Method 5
3. NMR Spectra 7
(*E*)-prop-1-en-1-ylbenzene, 2a 7
(*E*)-1-methyl-4-(prop-1-en-1-yl)benzene, 2b 9
(*E*)-1-methoxy-4-(prop-1-en-1-yl)benzene, 2c 10
(*E*)-1-fluoro-4-(prop-1-en-1-yl)benzene, 2d 12
(*E*)-1-methyl-3-(prop-1-en-1-yl)benzene, 2e 13
(*E*)-1-methyl-2-(prop-1-en-1-yl)benzene, 2f 14
(*E*)-1-methoxy-2-(prop-1-en-1-yl)benzene, 2g 15
(*E*)-Isosafrole, 2h 17
4. References 18

1. Experimental Details

1.1 General Experimental Details

All reagents were purchased from VWR were used without further purification. Solvents (C_6D_6 and CD_3CN) were dried using activated molecular sieves before use. All NMR data were collected from 400 or 500 MHz Bruker or Agilent machines at 298 K and referenced to residual protic solvent (deuterated acetonitrile). Unless otherwise stated, all experimental manipulations were carried out under an inert atmosphere using standard Schlenk line/glovebox techniques. 1,3,5-trimethoxybenzene was used as the internal standard to determine the spectroscopic yields. Experiments were performed in an M-Braun glove box under an argon or nitrogen atmosphere.

1.2 General Procedure

0.25 mmol equivalent of each substrate was added into a J-Young NMR tube containing a solution of 50 mol% of tetramethylammonium fluoride and between 15-20 mg of 1,3,5-trimethoxybenzene in dry deuterated acetonitrile. The reaction was allowed to proceed for 24 hours, the reaction was filtered, and the filtrate was analysed with NMR spectroscopy.

1.3 Scale up procedure of Allylbenzene, 2a.

Allylbenzene (850 µL, 6.42 mmol) was added to a suspension of tetramethylammonium fluoride (239 mg, 40 mol%) in anhydrous acetonitrile (13 mL). The reaction was stirred for 27 hours, solvents were removed *in vacuo.* The reaction was analysed by NMR spectroscopy with 0.1 mmol of 1,3,5-trimethoxybenzene (spectroscopic yield: 81%). The product was extracted in toluene and then washed three times with water (3 x 20 mL). The organic layer was dried with $MgSO_4$, filtered and solvents removed *in vacuo*, yielding (*E*)-prop-1-en-1-ylbenzene (356 mg, 47%) as a dark yellow oil.

1.4 Characterisation of products

(*E*)-prop-1-en-1-ylbenzene, **2a**

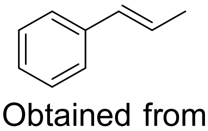

Obtained from the standard procedure with 100% spectroscopic yield with 40 mol% loading of Me_4NF.

^{1}H NMR (400 MHz, CD_3CN) δ 7.37 – 7.26 (m, 4H), 7.22 – 7.16 (m, 1H), 6.42 (d, *J* = 15.7 Hz, 1H), 6.33 – 6.23 (m, 1H), 1.88 – 1.80 (m, 3H).
^{13}C{^{1}H} NMR (101 MHz, CD_3CN) δ 138.5, 131.4, 129.1, 127.4, 126.3, 18.2.
Data is consistent with that obtained from a pure sample of *trans*-β-methyl styrene.

^{19}F{^{1}H} NMR (376 MHz, CD_3CN) δ −80.71 (s), −148.00 (t, DF_2^-)

(*E*)-1-methyl-4-(prop-1-en-1-yl)benzene, 2b

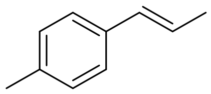

Obtained from the standard procedure with 81% conversion with 50 mol% loading of Me_4NF. 41% conversion was observed with 40 mol% loading of Me_4NF.

^{1}H NMR (400 MHz, CD_3CN) δ 7.19 – 7.12 (m, 4H), 6.37 (d, *J* = 16.3, 1H), 6.30 – 6.21 (m, 1H), 2.29 (s, 3H), 1.85-1.80 (m, 3H).
^{13}C{^{1}H} NMR (101 MHz, CD_3CN) δ 139.1, 138.9, 131.9, 129.4, 128.5, 127.4, 123.9, 21.4, 18.7
Data is concordant with previous literature.[1]

^{19}F{^{1}H} NMR (470 MHz, CD_3CN) δ -75.98 (s, 82%), -147.87 (t, 16%, DF_2^-), -147.96 (1%).

(*E*)-1-methoxy-4-(prop-1-en-1-yl)benzene, 2c

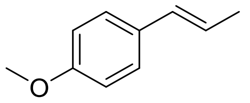

Obtained from the standard procedure with 100% spectroscopic yield with 40 mol% loading of Me_4NF.

^{1}H NMR (500 MHz, CD_3CN) δ 7.27 – 7.24 (m, 2H), 6.88 – 6.82 (m, 2H), 6.35 (d, *J* = 15.9, 1H), 6.13 – 6.10 (m, 1H), 3.74 (s, 3H), 1.82 – 1.78 (m, 3H).
^{13}C{^{1}H} NMR (101 MHz, CD_3CN) δ 159.8, 131.7, 131.2, 127.9, 124.3, 114.9, 55.9, 18.6.
Data is concordant with previous literature.[2]

^{19}F{^{1}H} NMR (471 MHz, CD_3CN) δ −80.91 (br, s, Me_4NF, 83%), −147.25 (t, DF_2^-, 17%).

(*E*)-1-fluoro-4-(prop-1-en-1-yl)benzene, 2d

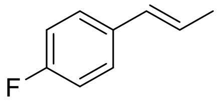

Obtained from the standard procedure with 100% spectroscopic yield with 40 mol% loading of Me_4NF.

^{1}H NMR (500 MHz, CD_3CN) δ 7.34 (dd, *J* = 8.5, 5.6, 2H), 7.02 (t, *J* = 8.7 Hz, 2H), 6.38 (d, *J* = 16.3 Hz, 1H), 6.26 – 6.15 (m, 1H), 1.82 (d, *J* = 6.1Hz, 3H).
^{13}C{^{1}H} NMR (126 MHz, CD_3CN) δ 161.8 (d, *J* = 243 Hz), 134.5 (d, *J* = 3.3 Hz), 130.6, 128.4 (d, *J* = 8.3 Hz), 126.7 (d, *J* = 2.6 Hz), 116.1 (d, *J* = 21.6 Hz), 18.6.
^{19}F{^{1}H} NMR (470 MHz, CD_3CN) δ −76.44(br s, 22%), −117.49 (m, 8%), −117.65 (m, 66%), −147.85 (t, DF_2^-, 4%).

Data is concordant with previous literature.[1]

(*E*)-1-methyl-3-(prop-1-en-1-yl)benzene, 2e

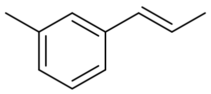

Obtained from the standard procedure with 75% spectroscopic yield with 40 mol% loading of Me_4NF.

^{1}H NMR (400 MHz, CD_3CN) δ 7.25 – 6.97 (m, 4H), 6.38 (d, *J* = 15. 7 Hz, 1H), 6.30-6.22 (m, 1H), 2.29 (s, 3H), 1.85-1.80 (m, 3H).
^{13}C{^{1}H} NMR (101 MHz, CD_3CN) δ 139.1, 138.9, 131.9, 128.5, 127.7, 127.4, 126.5, 123.9, 21.4, 18.7.
Data is concordant with previous literature.[3]

(*E*)-1-methyl-2-(prop-1-en-1-yl)benzene, **2f**

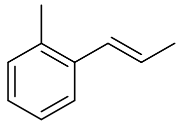

Obtained from the standard procedure with 46% spectroscopic yield with 40 mol% loading of Me_4NF.

^{1}H NMR (400 MHz, CD_3CN) δ 7.41-7.12 (m, 4H), 6.64 (dt, *J* = 15.7, 1.7 Hz, 1H), 6.19 – 6.11 (m, 1H), 2.29 (s, 3H), 1.88 – 1.83 (m, 3H).
^{13}C{^{1}H} NMR (101 MHz, CD_3CN) δ 137.3, 135.8, 130.7, 129.6, 127.3, 127.0, 126.2, 125.8, 19.9, 19.4.
Data is concordant with previous literature.[1]

(*E*)-1-methoxy-2-(prop-1-en-1-yl)benzene, 2g

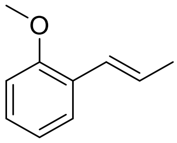

Obtained from the standard procedure with 100% spectroscopic yield with 40 mol% loading of Me_4NF.

^{1}H NMR (500 MHz, CD_3CN) δ 7.39 (dd, J = 7.6, 1.7 Hz, 1H), 7.18 (ddd, *J* = 8.8, 7.4, 1.7 Hz, 1H), 6.93 – 6.85 (m, 2H), 6.68 (dt, *J* = 15.9, 1.8 Hz, 1H), 6.28 – 6.18 (m, 1H), 3.79 (d, *J* = 7.8 Hz, 3H), 1.87 – 1.80 (m, 3H).
^{13}C{^{1}H} NMR (101 MHz, CD_3CN) δ 157.3, 129.0, 127.7, 127.2, 126.6, 121.6, 120.9, 112.0, 56.0, 19.1.
Data is concordant with previous literature.[1]

^{19}F{^{1}H} NMR (376 MHz, CD_3CN) δ −80.27 (s, 82%), −147.36 (t, DF_2^-, 18%)

(*E*)-Isosafrole, 2h

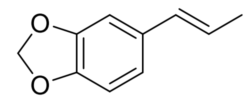

Obtained from the standard procedure with 100% spectroscopic yield with 40 mol% loading of Me_4NF.

^{1}H NMR (400 MHz, CD_3CN) δ 6.94 – 6.69 (m, 3H), 6.30 (m, 1H), 6.14 – 6.02 (m, 1H), 5.90 (s, 2H), 1.84 – 1.75 (m, 3H).
^{13}C{^{1}H} NMR (101 MHz, CD_3CN) δ 149.1, 147.6, 133.5, 131.4, 124.9, 121.1, 109.0, 106.0, 102.2, 18.5.
Data is concordant with previous literature.[4]

^{19}F{^{1}H} NMR (376 MHz, CD_3CN) δ −78.16 (br, s, 89%), −147.45 (t, 11%).

2. Kinetic Data

2.1 General Method

Reaction monitoring experiments were conducted following the general procedure outlined in section **1.2**. Any undissolved tetramethylammonium fluoride was allowed to settle before conducting ^{19}F{^{1}H} NMR reaction monitoring, however this could account for the unstable integration values observed in charts **S3** and **S4**.

Chart S1. Consumption of allylbenzene as a function of time collected using ^{1}H NMR spectroscopy. Reaction conditions: 0.5 mmol allylbenzene, 0.5 mL CD_3CN, 40 mol% Me_4NF, room temperature.

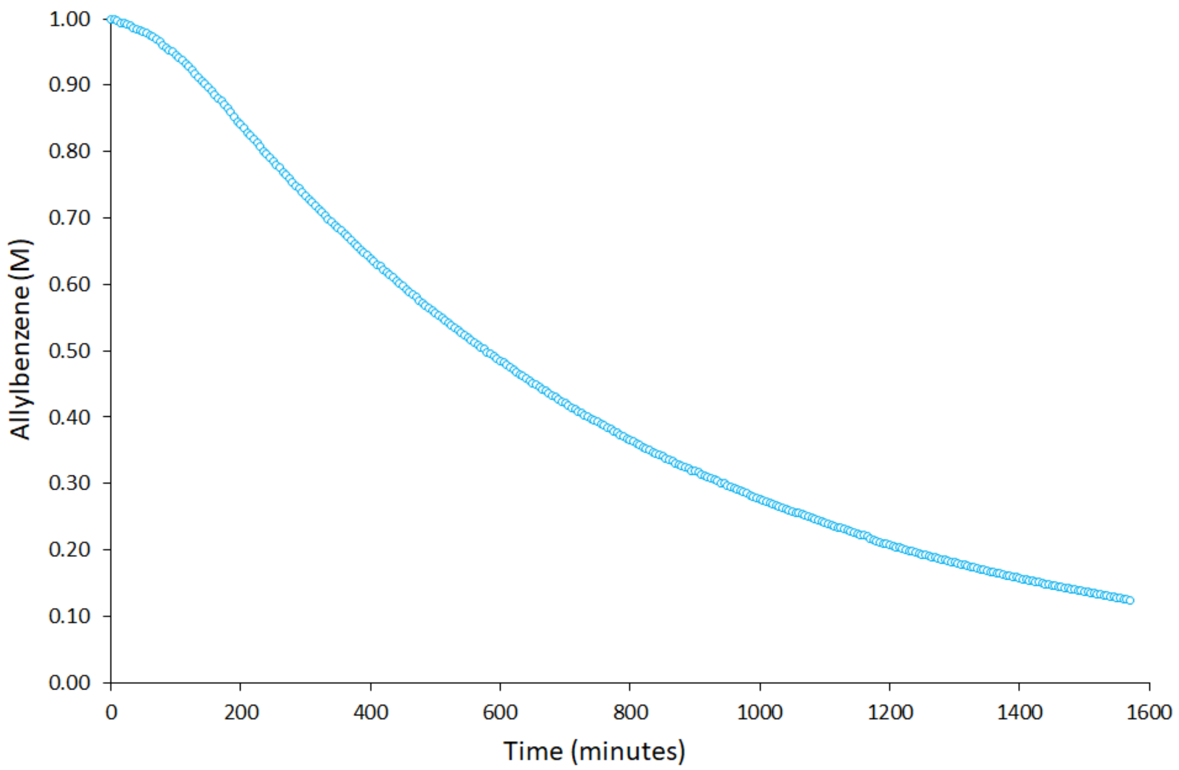

Chart S2. Pseudo 1st order plot showing the loss of allylbenzene.

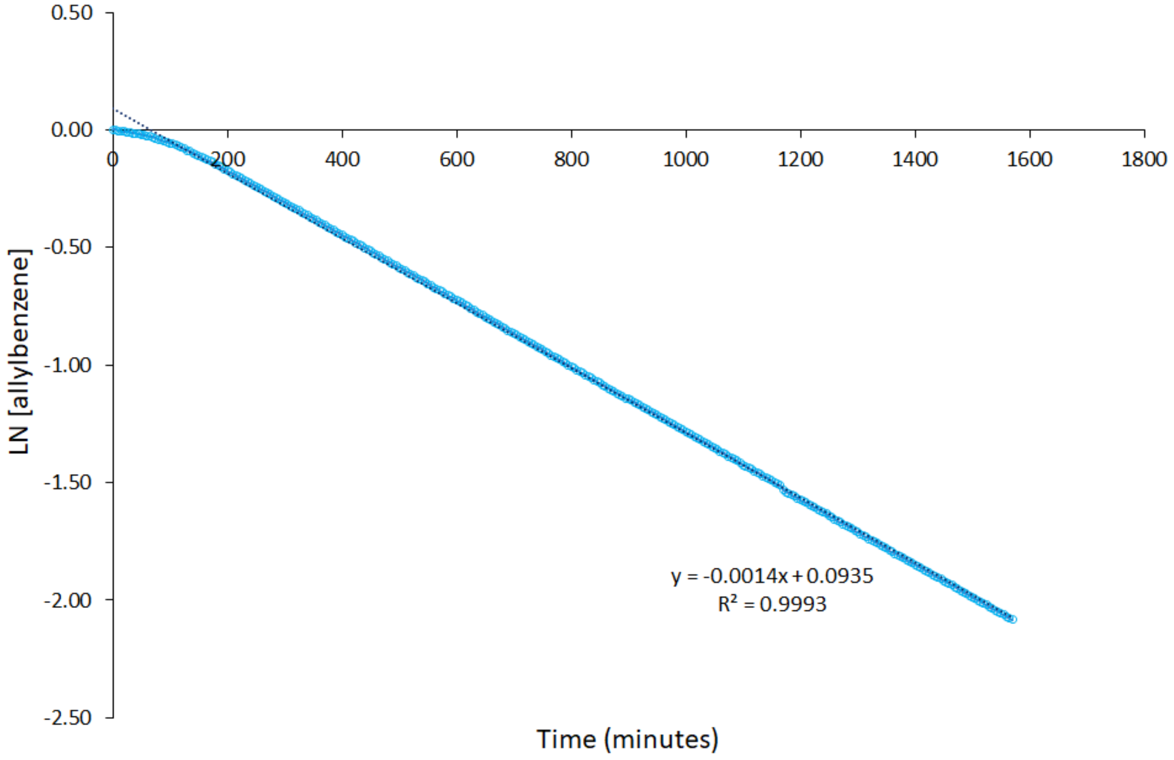

3. NMR Spectra

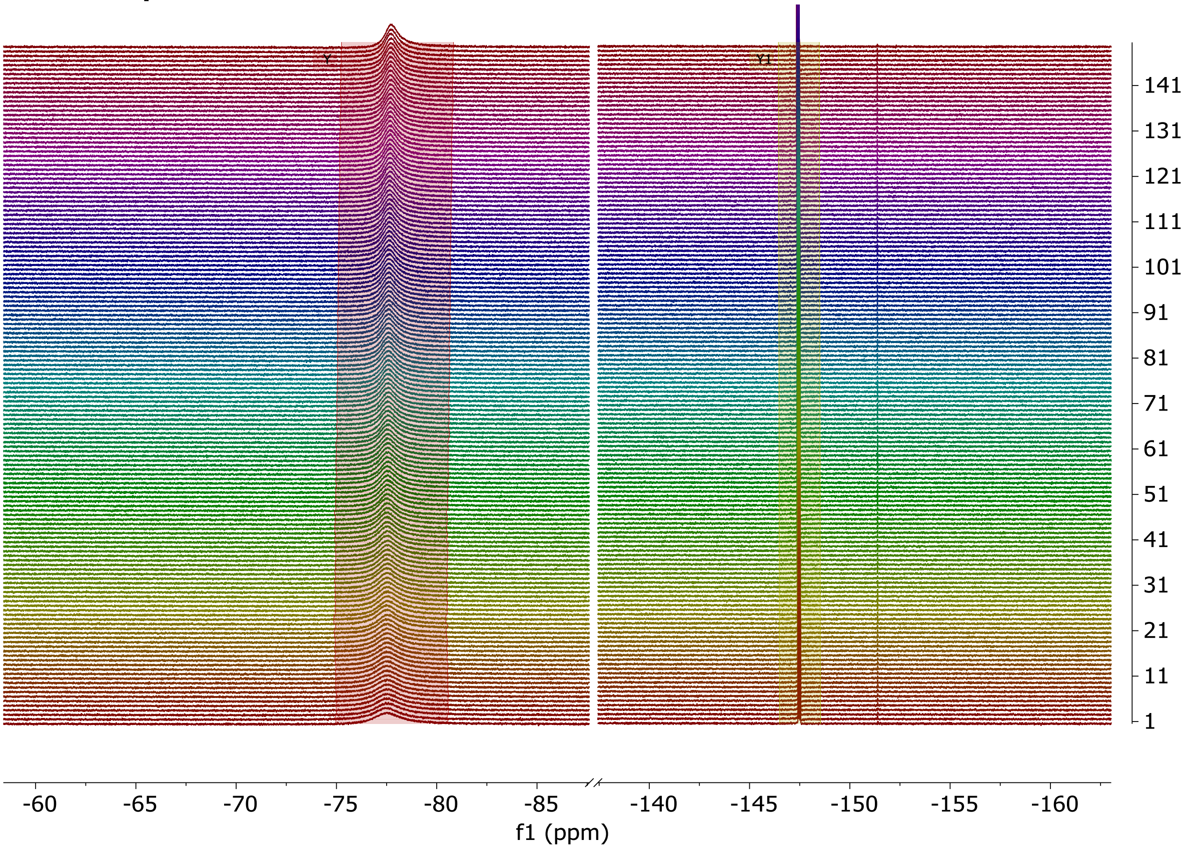

Figure S1. Superimposed $^{19}F\{^1H\}$ spectra for the isomerization of allylbenzene (0.25 mmol) with Me_4NF (40 mol%) in CD_3CN. Red highlight (Me_4NF) and yellow highlight (DF_2^-).

(*E*)-prop-1-en-1-ylbenzene, 2a

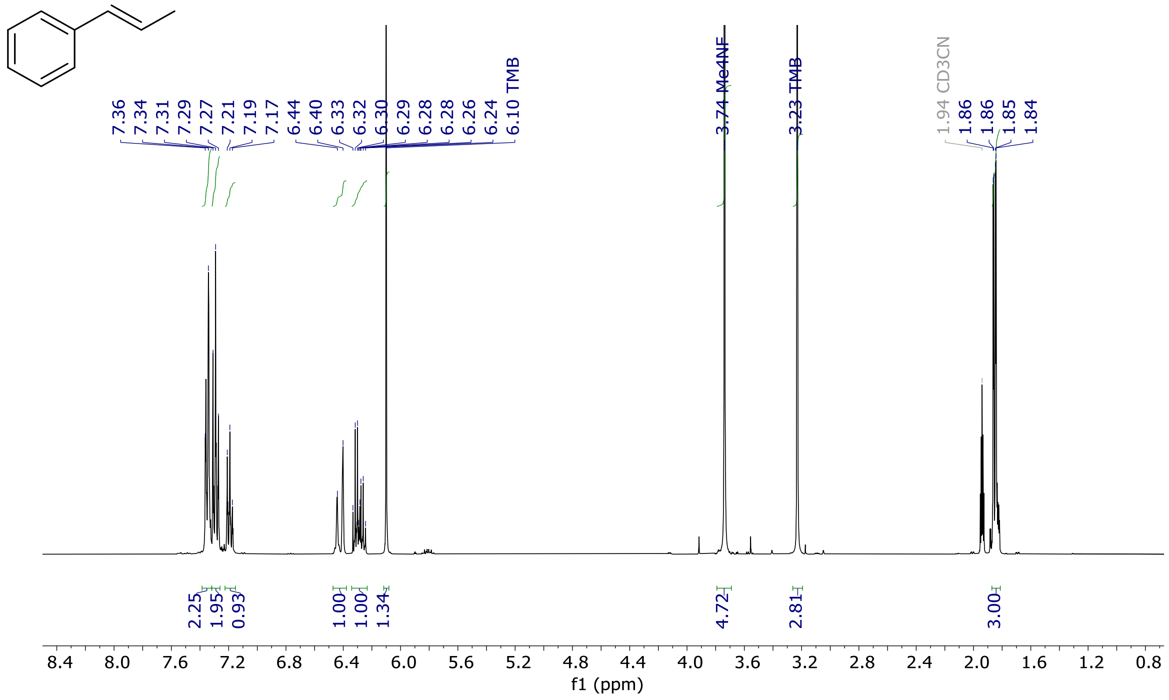

Figure S2. ^1H NMR (400 MHz, CD_3CN) of (*E*)-prop-1-en-1-ylbenzene, **2a**.

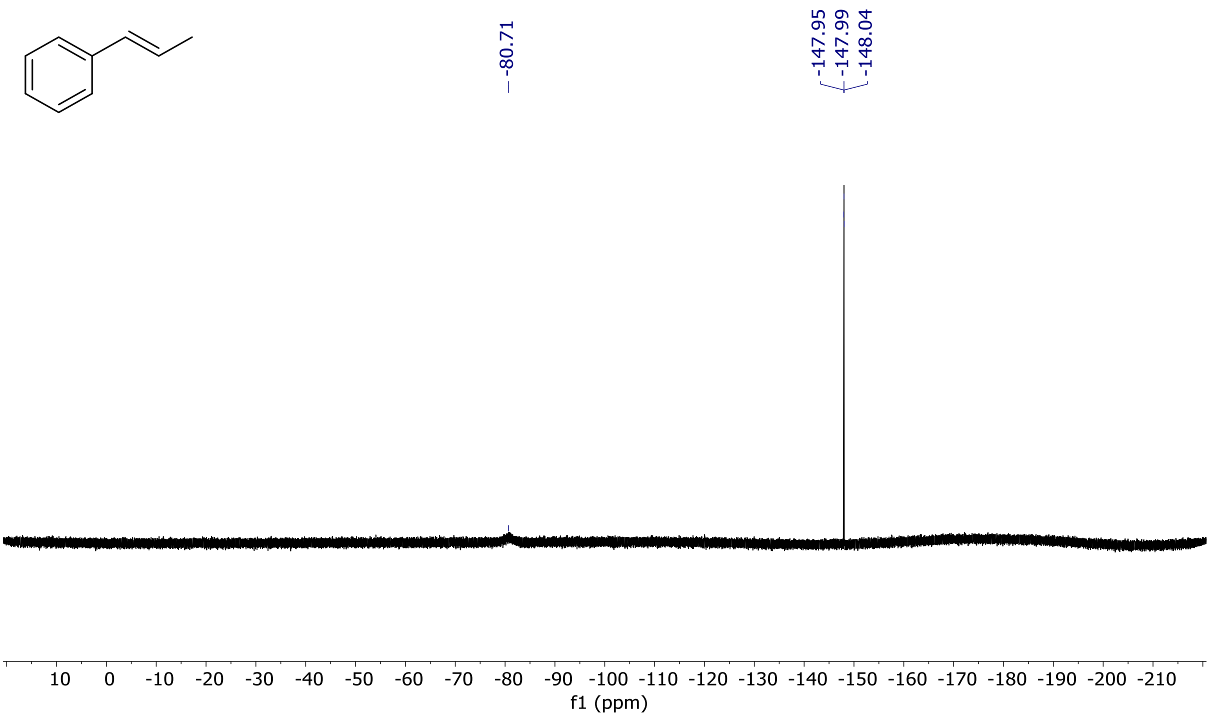

Figure S3. $^{19}F\{^1H\}$ NMR (376 MHz, CD_3CN) of (*E*)-prop-1-en-1-ylbenzene, **2a**.

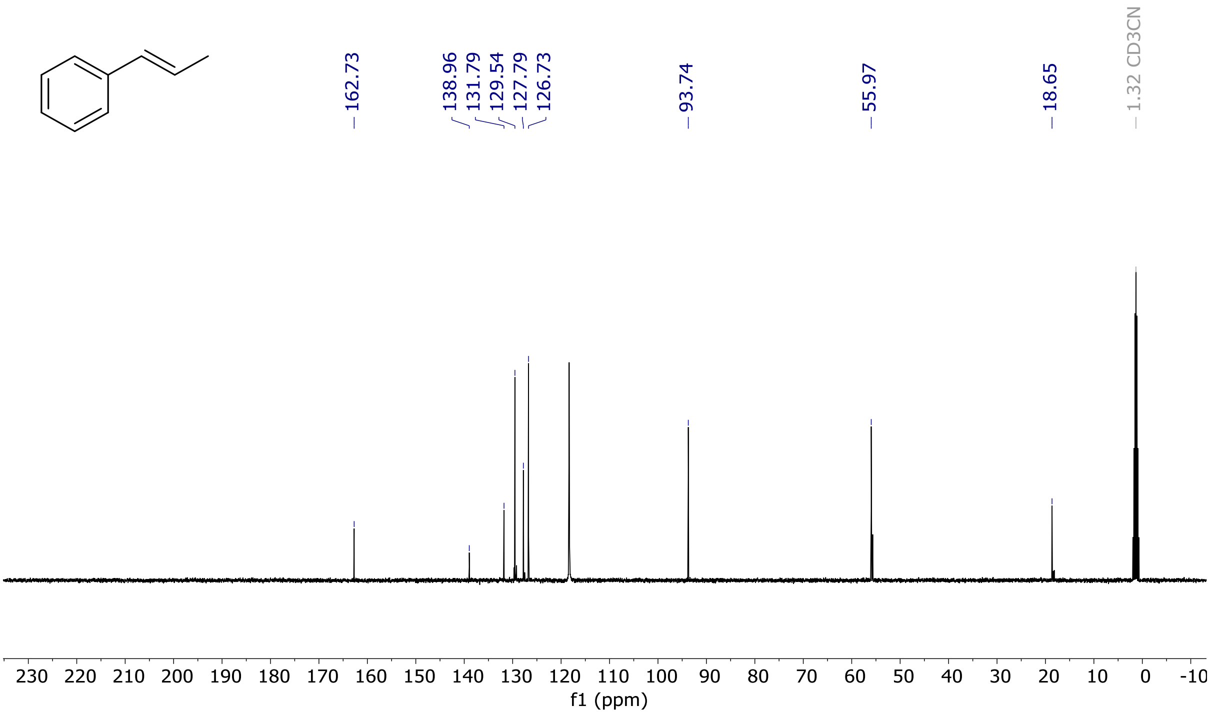

Figure S4. $^{13}C\{^1H\}$ NMR (101 MHz, CD_3CN) of (*E*)-prop-1-en-1-ylbenzene, **2a**.

(*E*)-1-methyl-4-(prop-1-en-1-yl)benzene, 2b

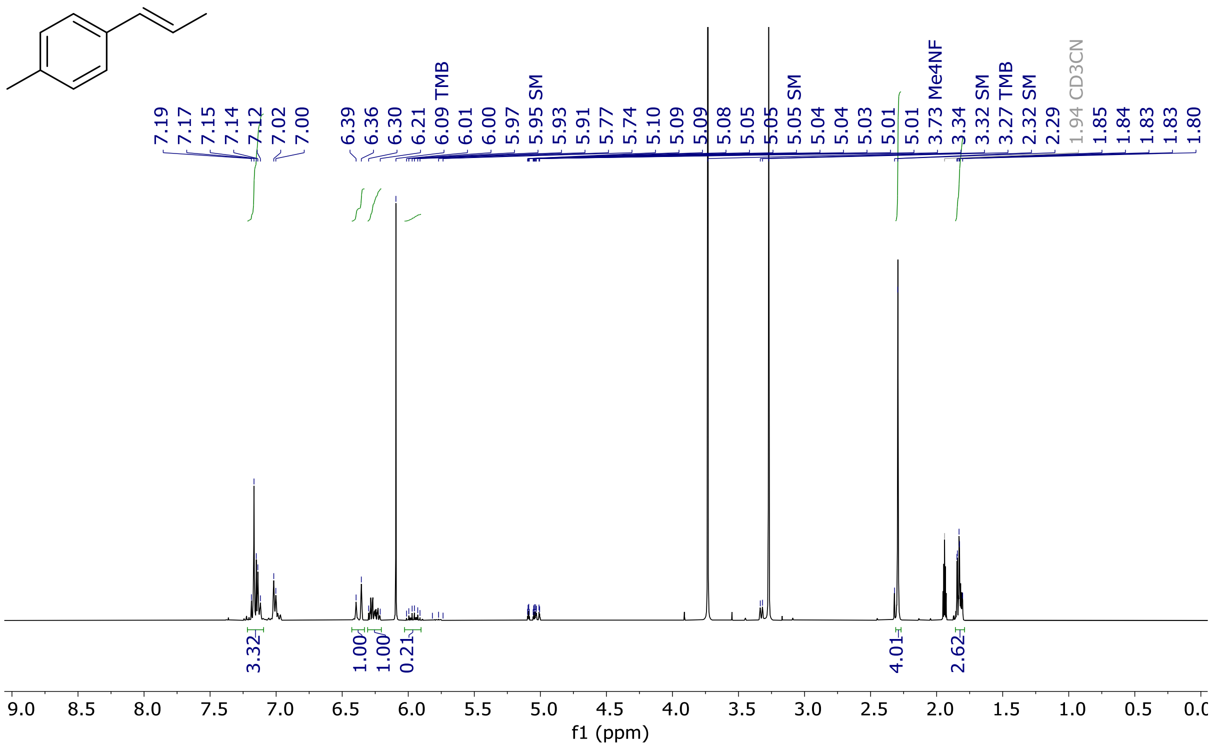

Figure S5. ^{1}H NMR (400 MHz, CD_3CN) of (*E*)-1-methyl-4-(prop-1-en-1-yl)benzene, **2b**.

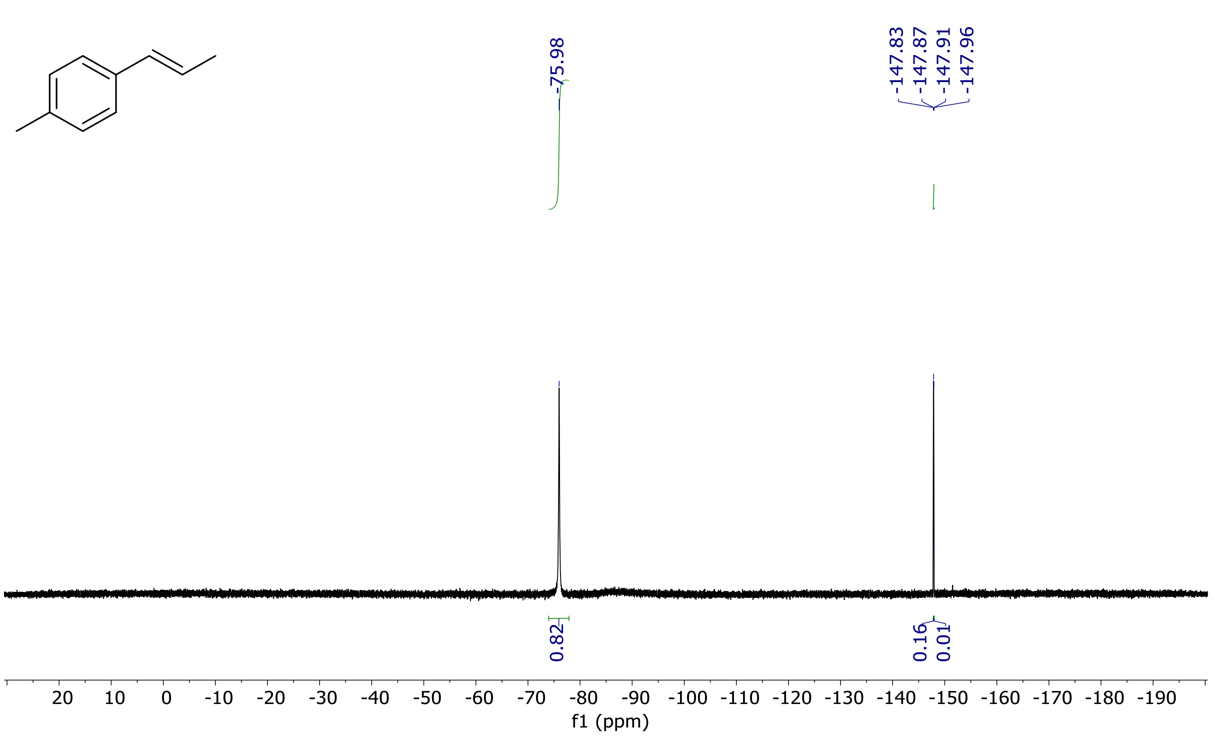

Figure S6. $^{19}F\{^{1}H\}$ NMR (471 MHz, CD_3CN) of (*E*)-1-methyl-4-(prop-1-en-1-yl)benzene, **2b**.

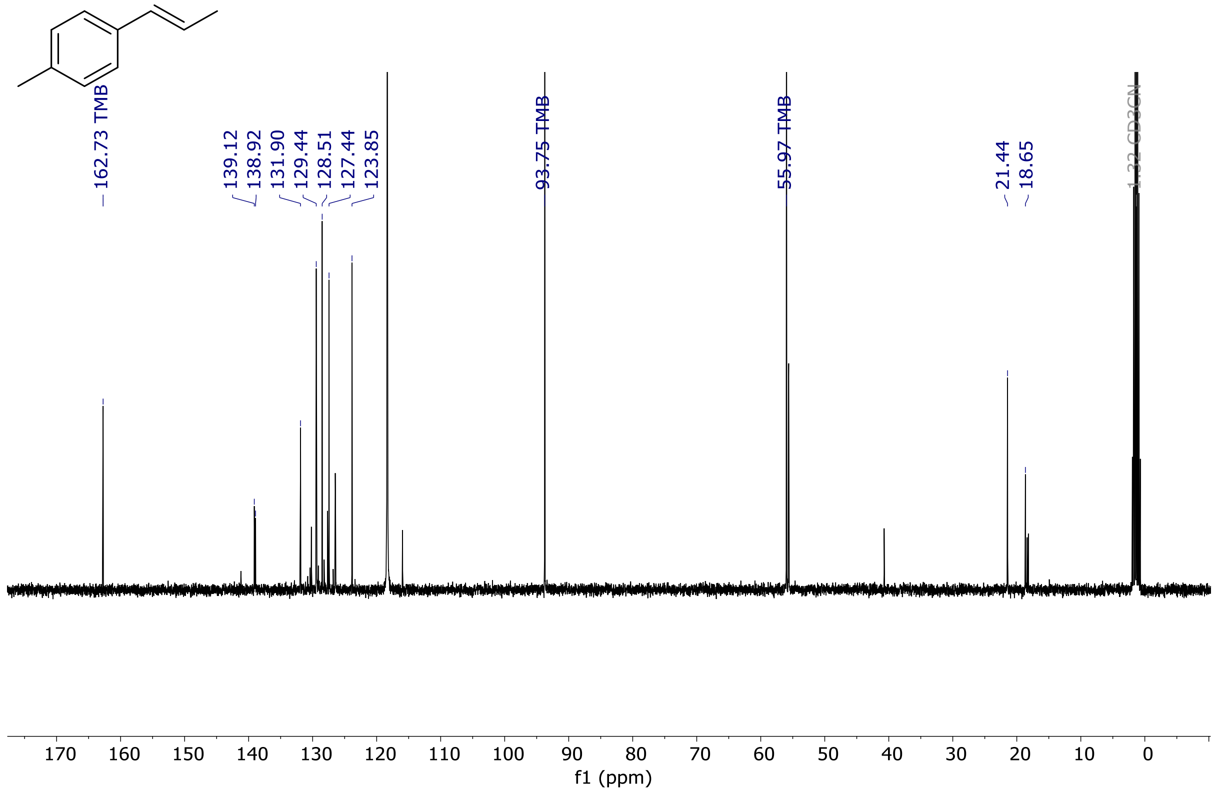

Figure S7. $^{13}C\{^{1}H\}$ NMR (101 MHz, CD_3CN) of (*E*)-1-methyl-4-(prop-1-en-1-yl)benzene, **2b**.

(*E*)-1-methoxy-4-(prop-1-en-1-yl)benzene, 2c

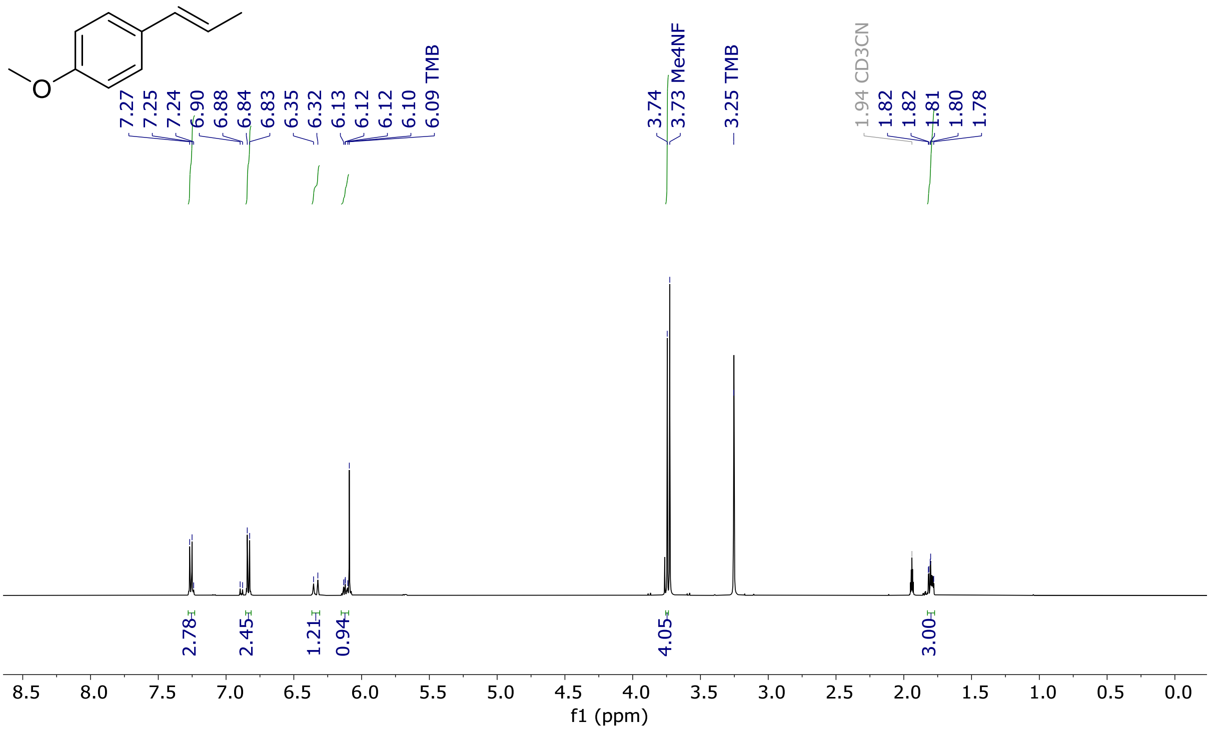

Figure S8. ^{1}H NMR (500 MHz, CD_3CN) of (*E*)-1-methoxy-4-(prop-1-en-1-yl)benzene, **2c**.

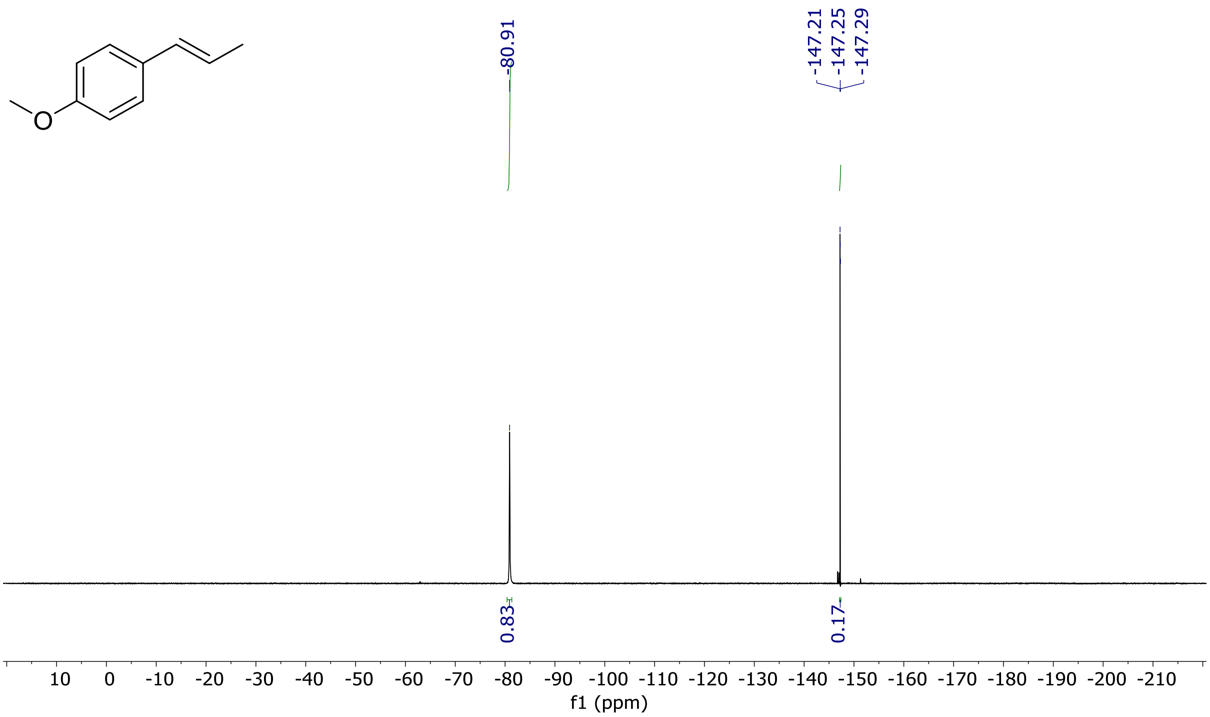

Figure S9. $^{19}F\{^{1}H\}$ NMR (471 MHz, CD_3CN) of (*E*)-1-methoxy-4-(prop-1-en-1-yl)benzene, **2c**.

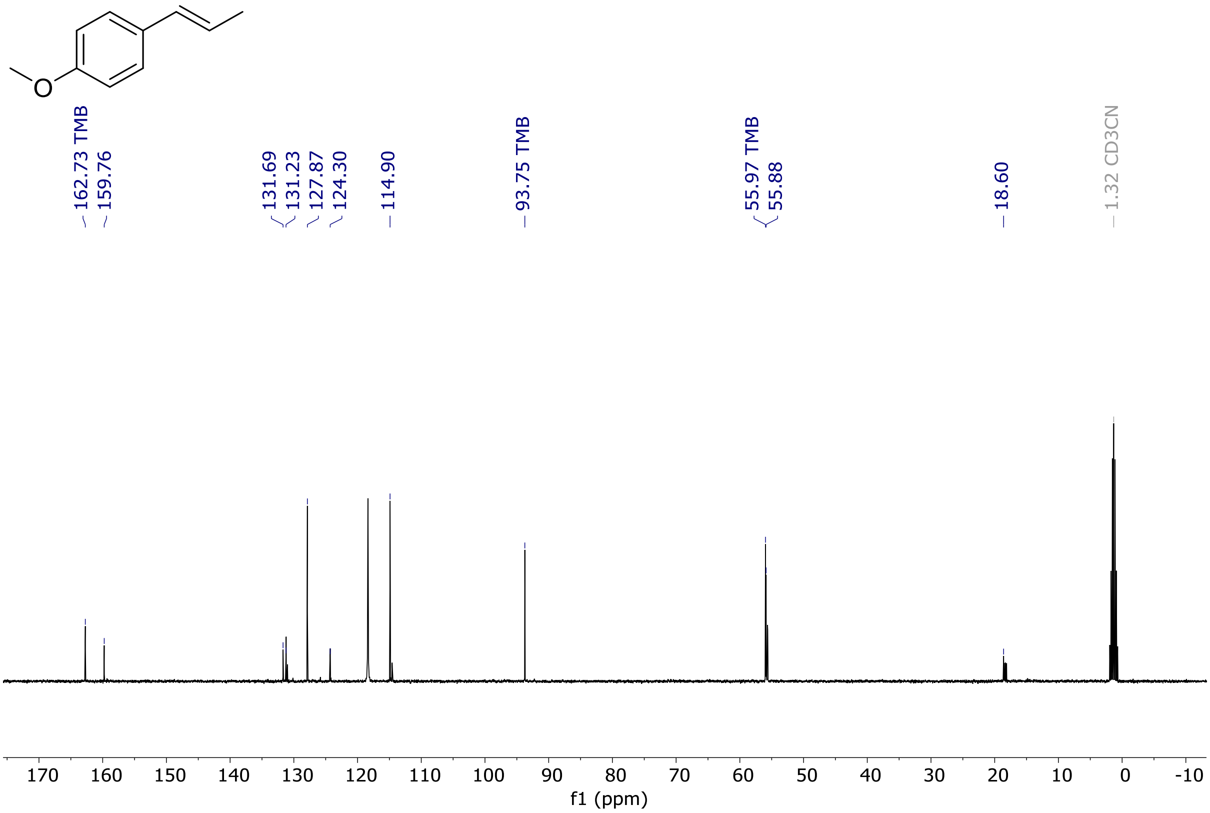

Figure S10. $^{13}C\{^{1}H\}$ NMR (101 MHz, CD_3CN) of (*E*)-1-methoxy-4-(prop-1-en-1-yl)benzene, **2c**.

(*E*)-1-fluoro-4-(prop-1-en-1-yl)benzene, 2d

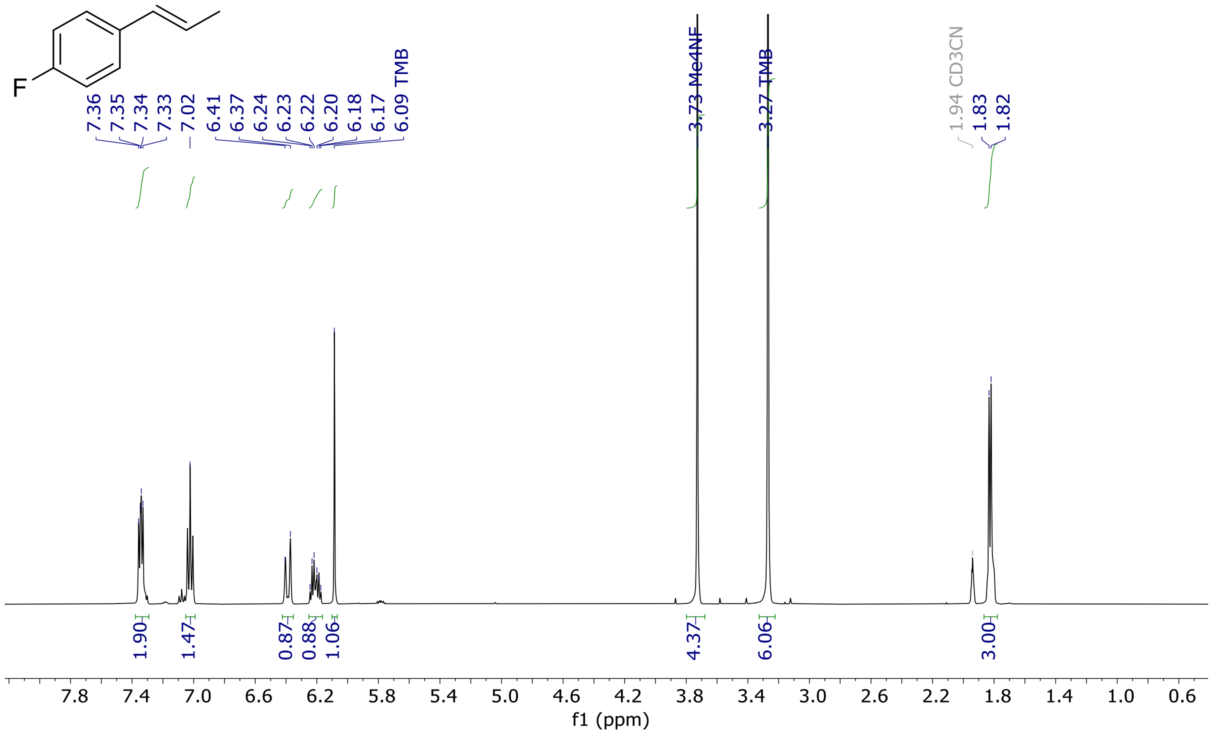

Figure S11. ^{1}H NMR (500 MHz, CD_3CN) of (*E*)-1-fluoro-4-(prop-1-en-1-yl)benzene, **2d**.

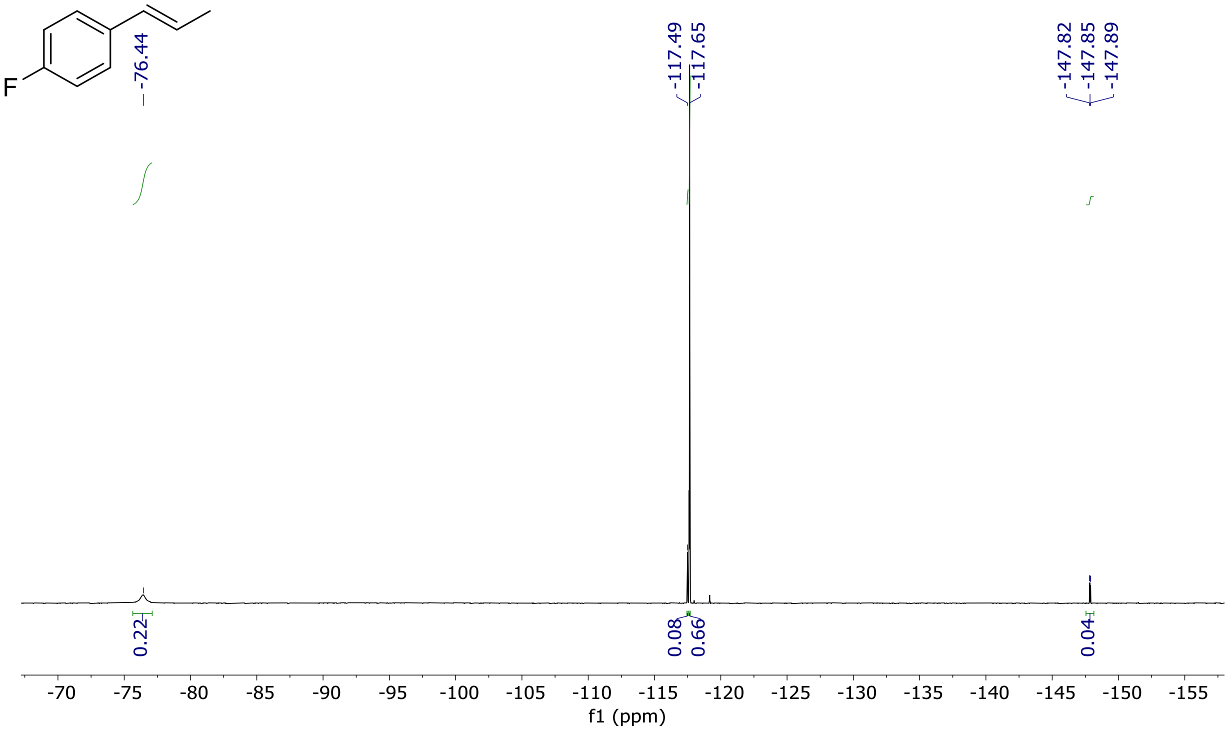

Figure S12. $^{19}F\{^{1}H\}$ NMR (470 MHz, CD_3CN) of (*E*)-1-fluoro-4-(prop-1-en-1-yl)benzene, **2d**.

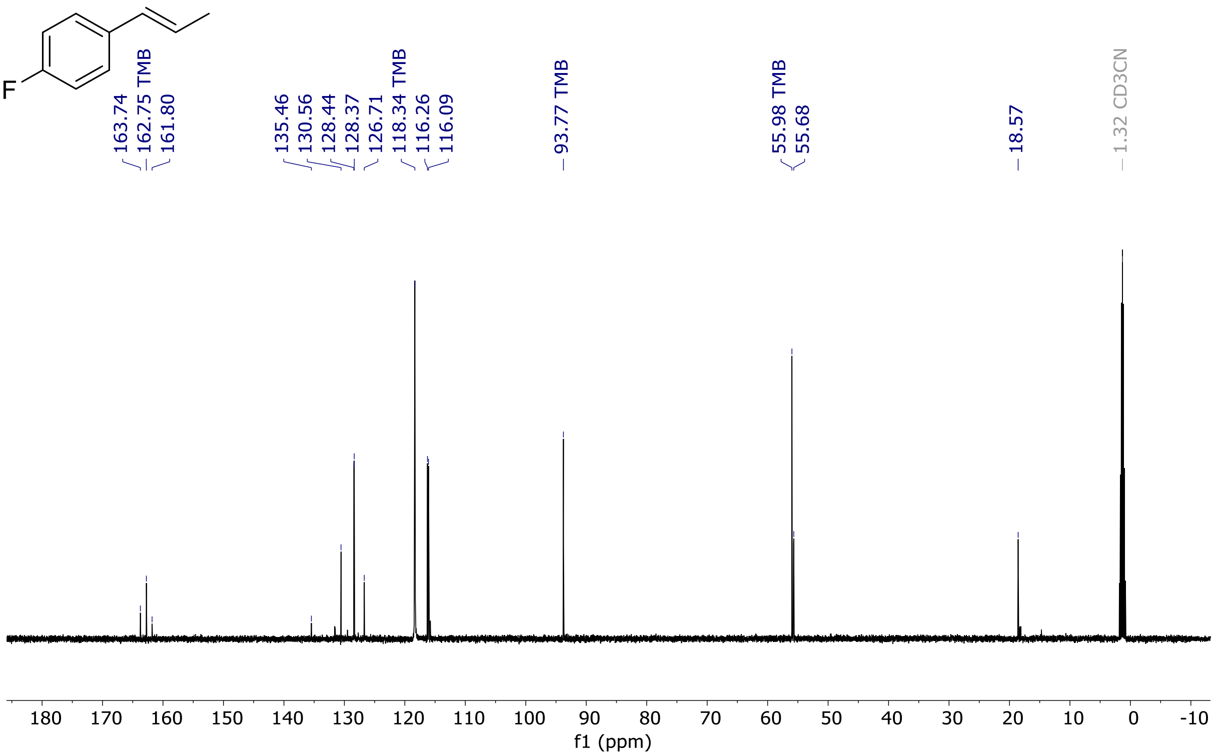

Figure S13. $^{13}C\{^{1}H\}$ NMR (126 MHz, CD_3CN) of (*E*)-1-fluoro-4-(prop-1-en-1-yl)benzene, **2d**.

(*E*)-1-methyl-3-(prop-1-en-1-yl)benzene, 2e

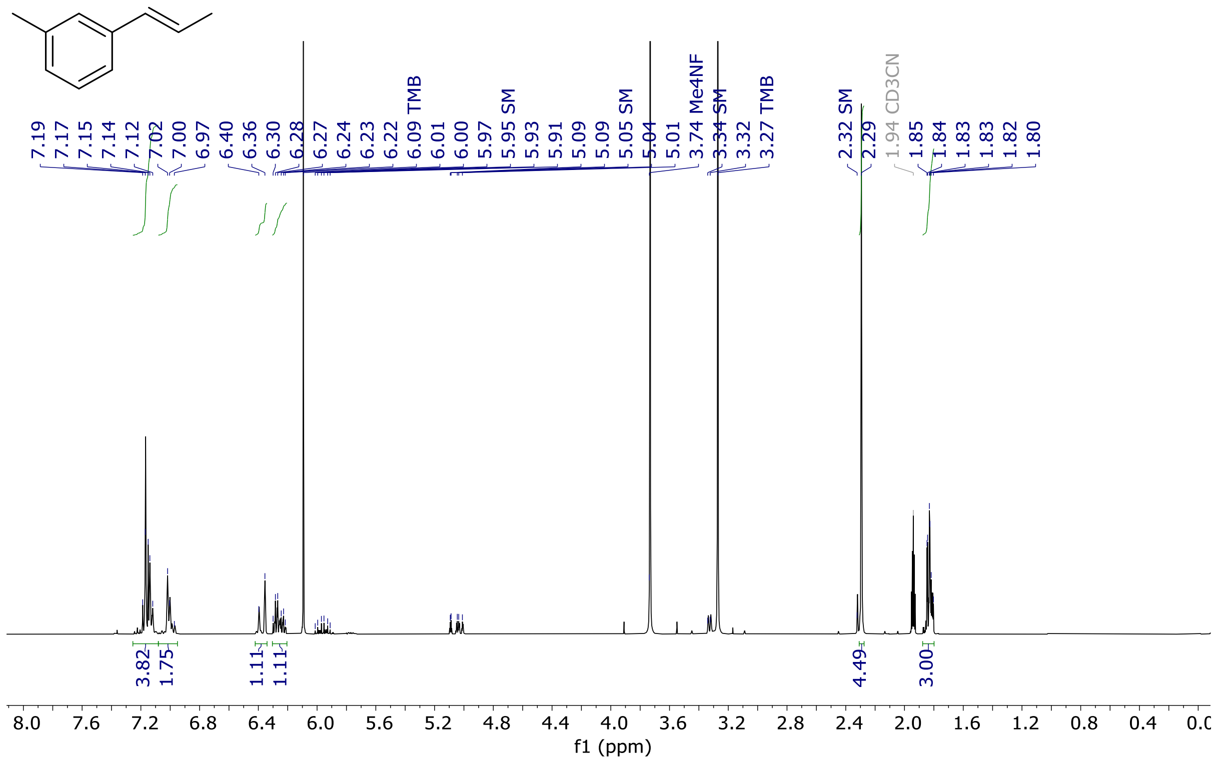

Figure S14. ^{1}H NMR (400 MHz, CD_3CN) of (*E*)-1-methyl-3-(prop-1-en-1-yl)benzene, **2e**.

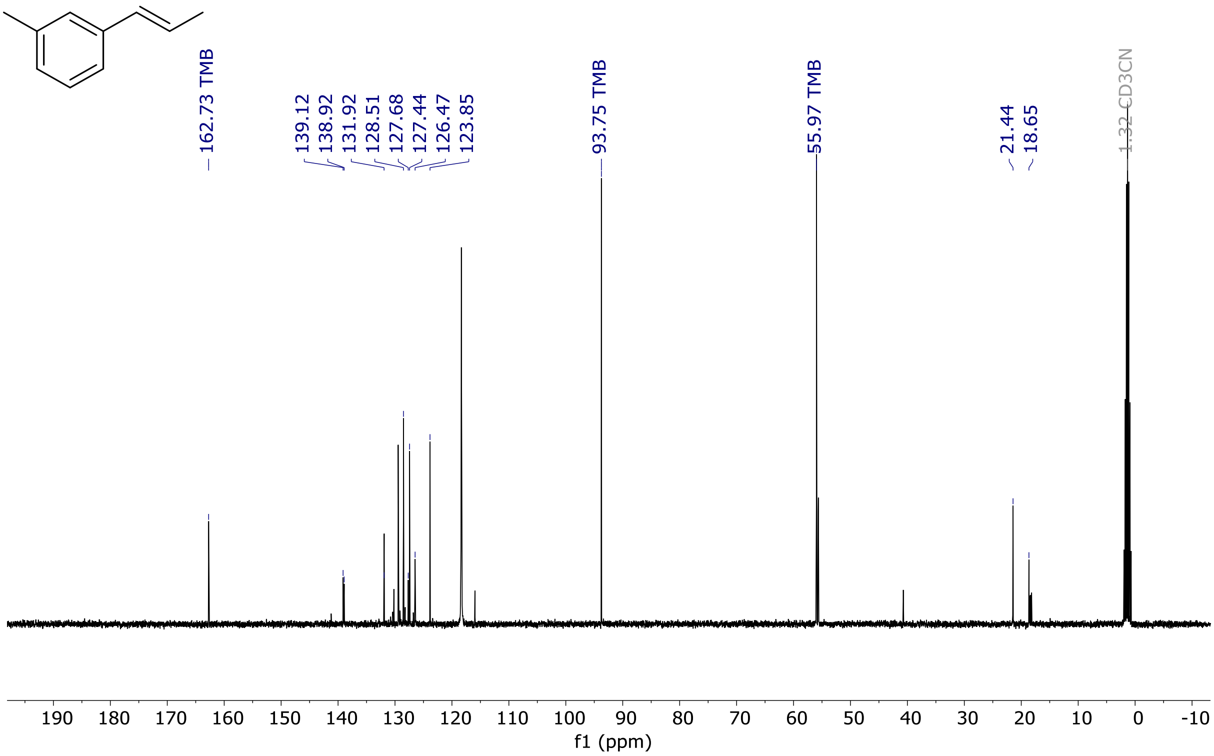

Figure S15. $^{13}C\{^{1}H\}$ NMR (101 MHz, CD_3CN) of (*E*)-1-methyl-3-(prop-1-en-1-yl)benzene, **2e**.

(*E*)-1-methyl-2-(prop-1-en-1-yl)benzene, 2f

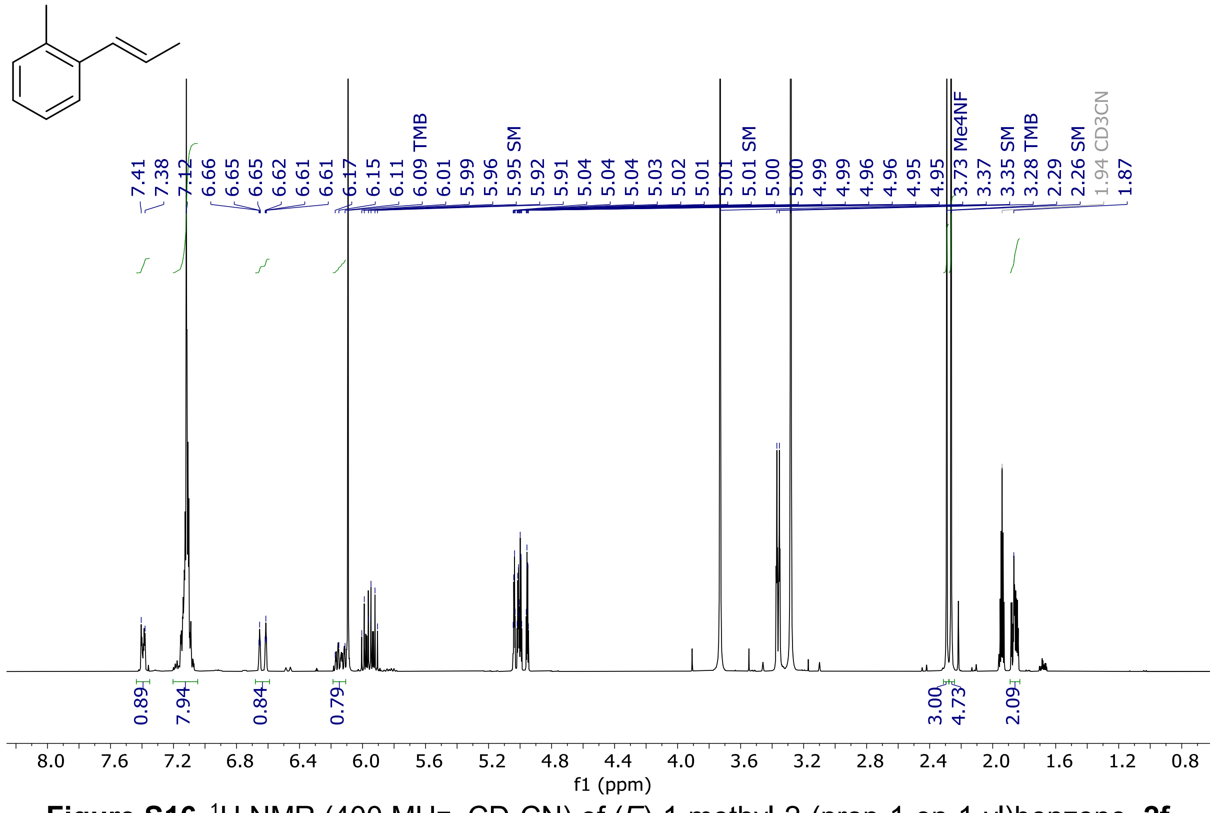

Figure S16. ^{1}H NMR (400 MHz, CD_3CN) of (*E*)-1-methyl-2-(prop-1-en-1-yl)benzene, **2f**.

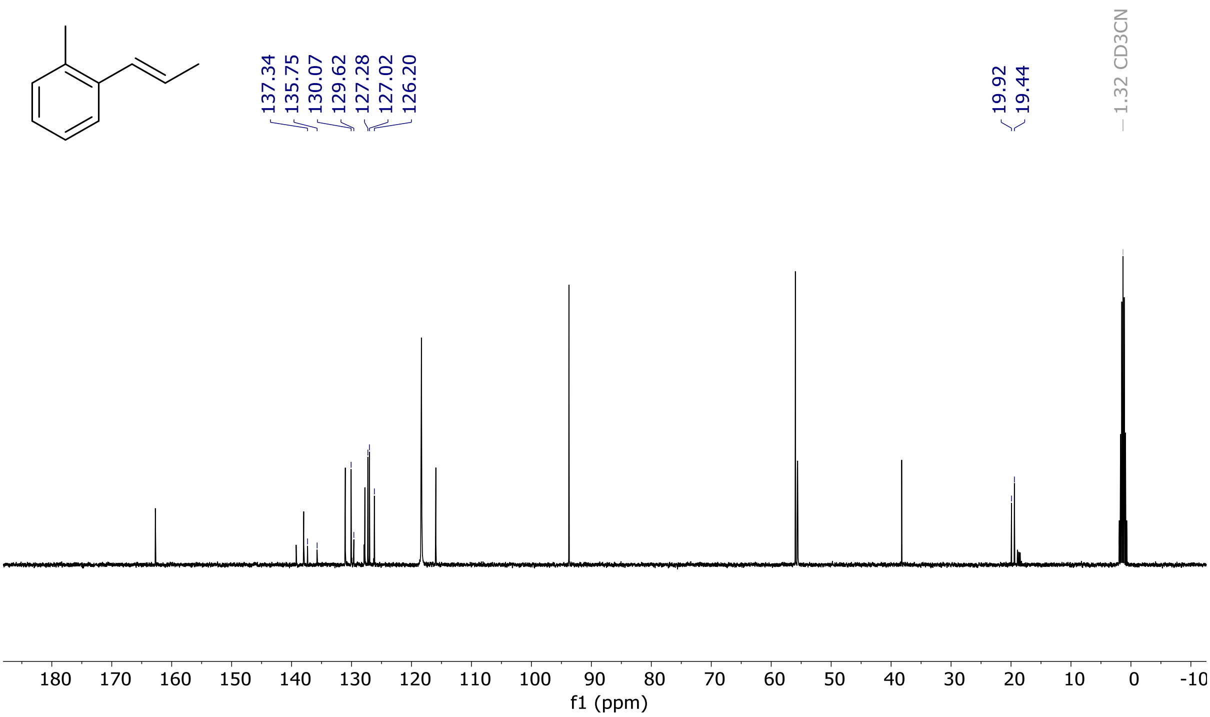

Figure S17. $^{13}C\{^{1}H\}$ NMR (101 MHz, CD_3CN) of (*E*)-1-methyl-2-(prop-1-en-1-yl)benzene, **2f**.

(*E*)-1-methoxy-2-(prop-1-en-1-yl)benzene, 2g

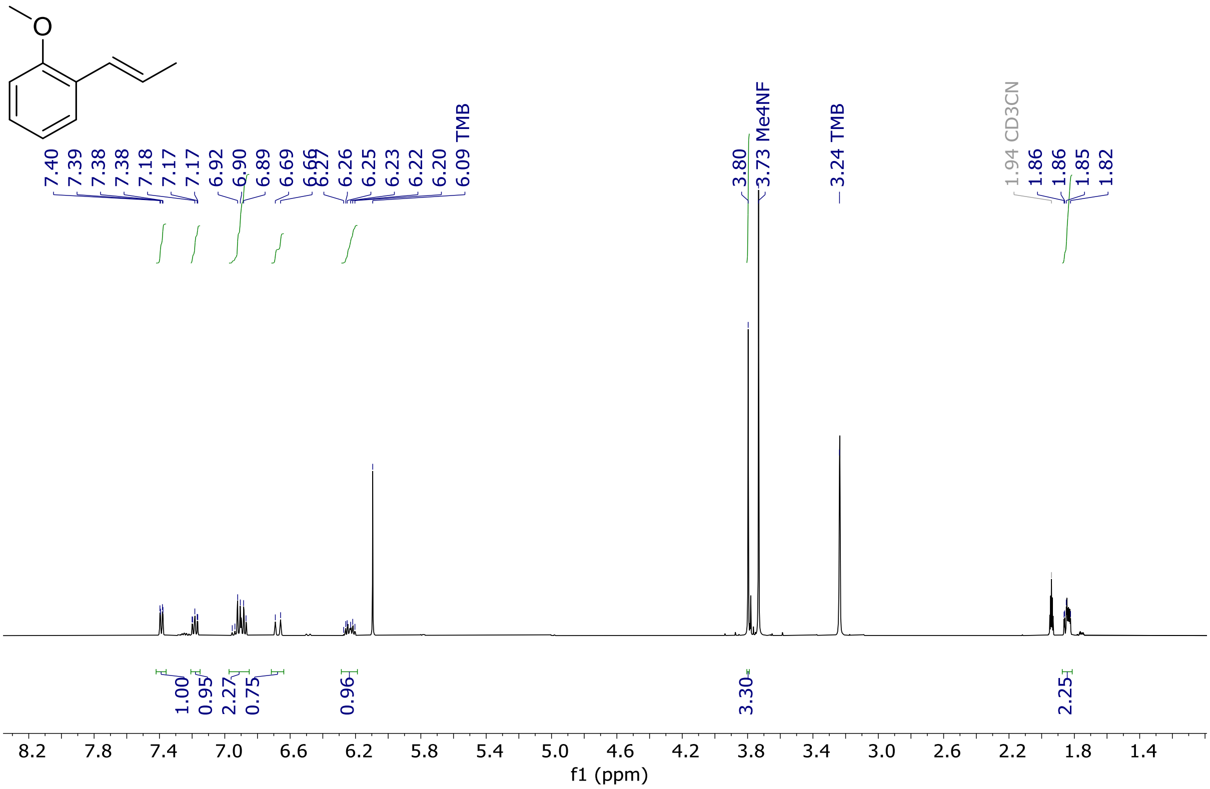

Figure S18. ^{1}H NMR (500 MHz, CD_3CN) of (*E*)-1-methoxy-2-(prop-1-en-1-yl)benzene, **2g**.

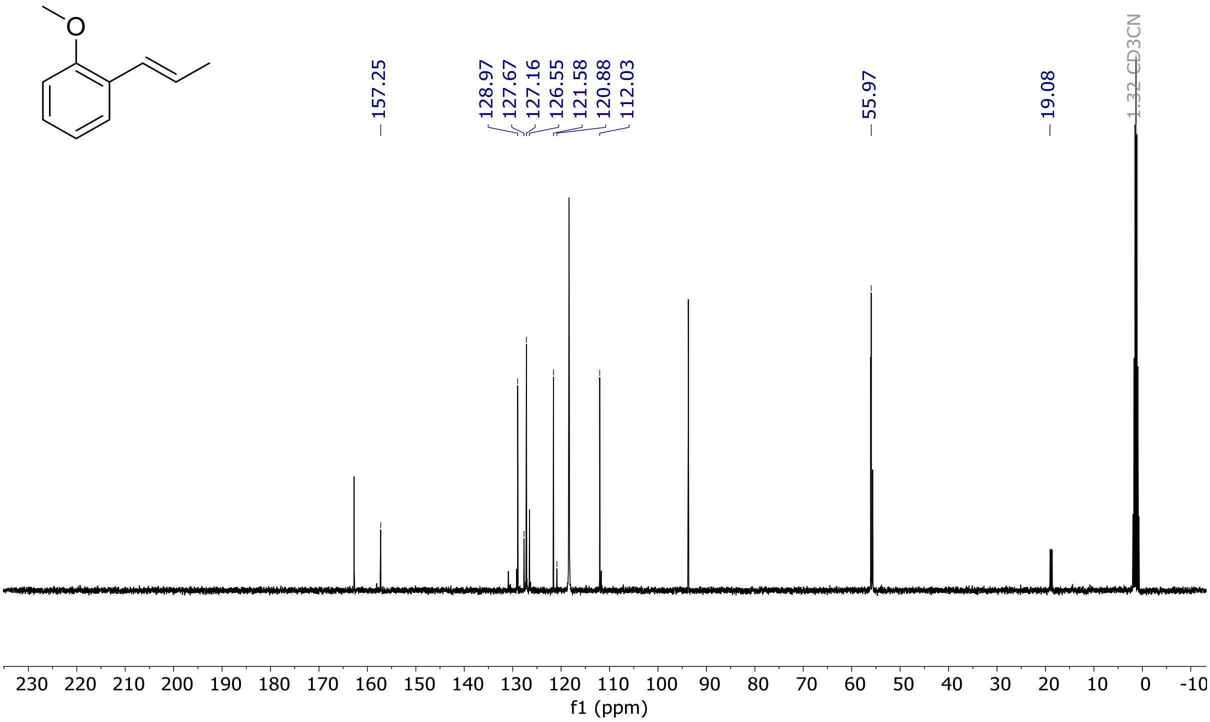

Figure S19. $^{19}F\{^{1}H\}$ NMR (MHz, CD_3CN) of (*E*)-1-methoxy-2-(prop-1-en-1-yl)benzene, **2g**.

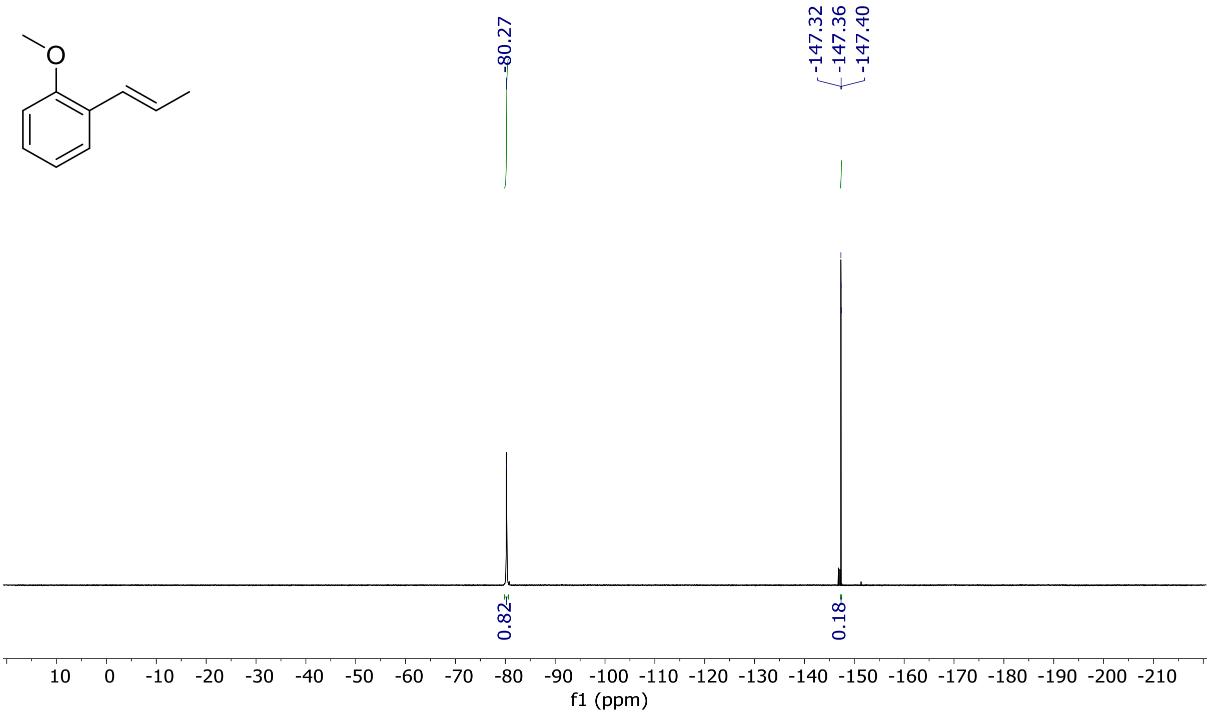

Figure S20. $^{13}C\{^{1}H\}$ NMR (101 MHz, CD_3CN) of (*E*)-1-methoxy-2-(prop-1-en-1-yl)benzene, **2g**.

(*E*)-Isosafrole, 2h

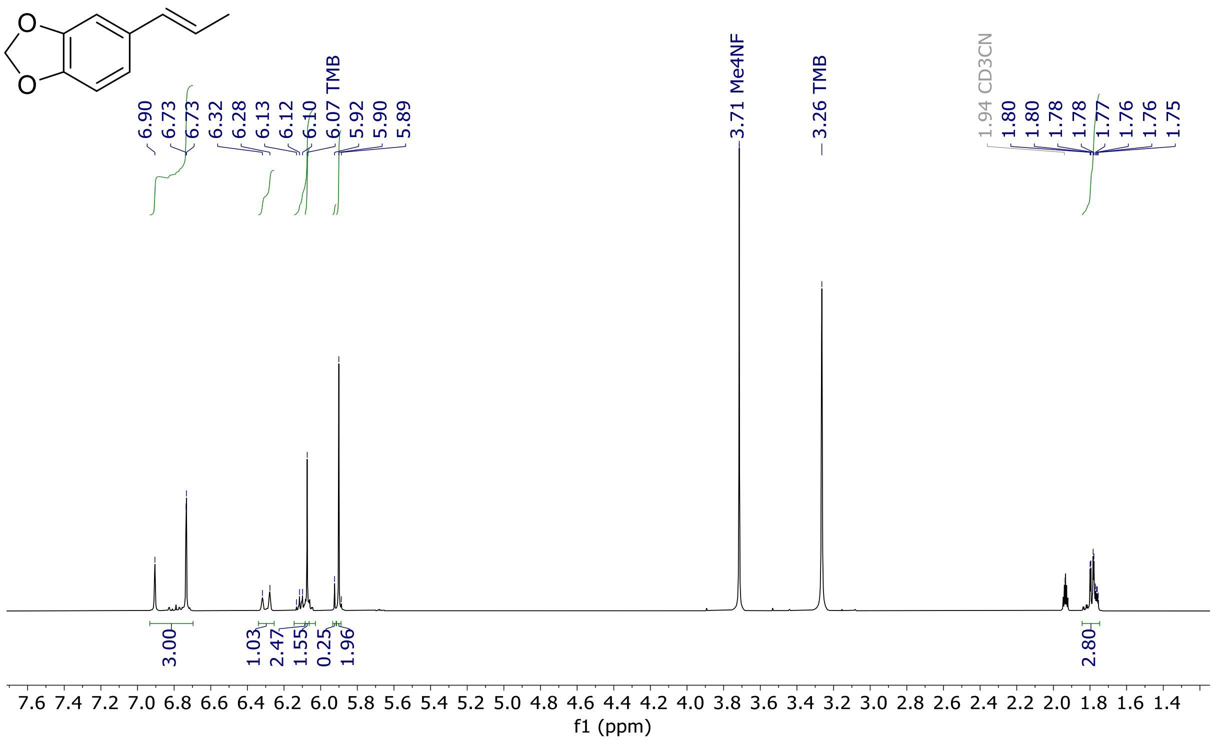

Figure S21. ^{1}H NMR (400 MHz, CD_3CN) of (*E*)-Isosafrole, **2h**.

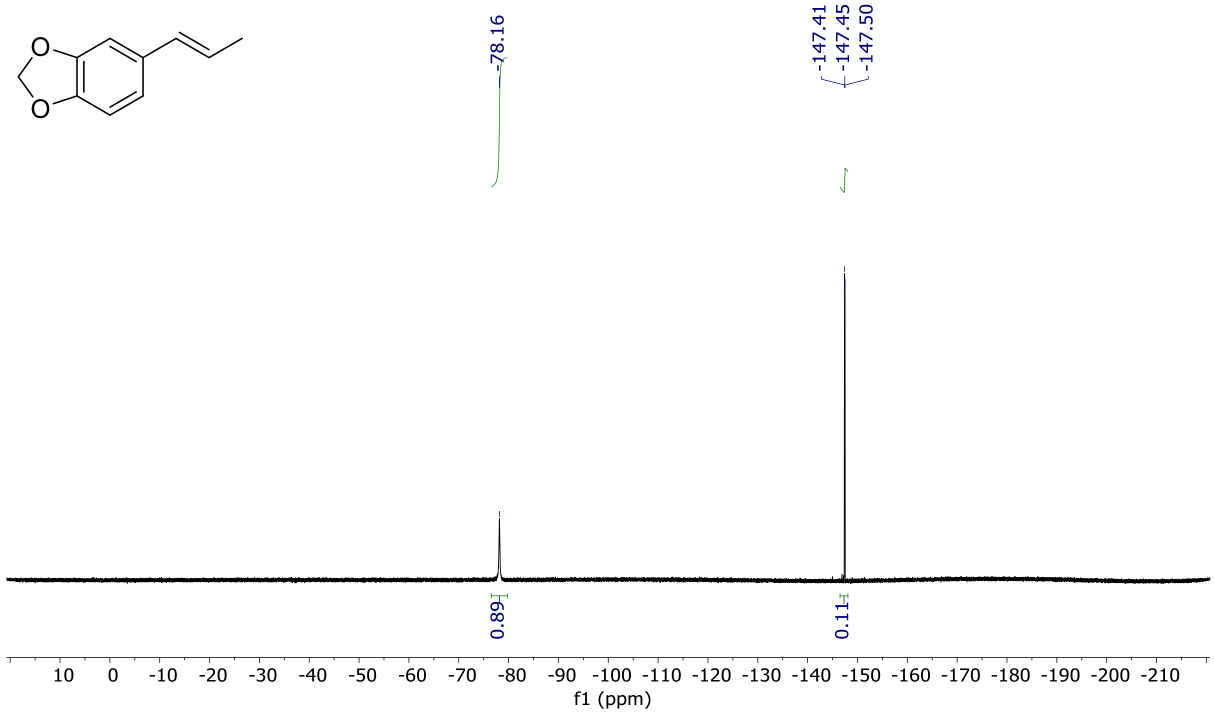

Figure S22. $^{19}F\{^{1}H\}$ NMR (376 MHz, CD_3CN) of (*E*)-Isosafrole, **2h**.

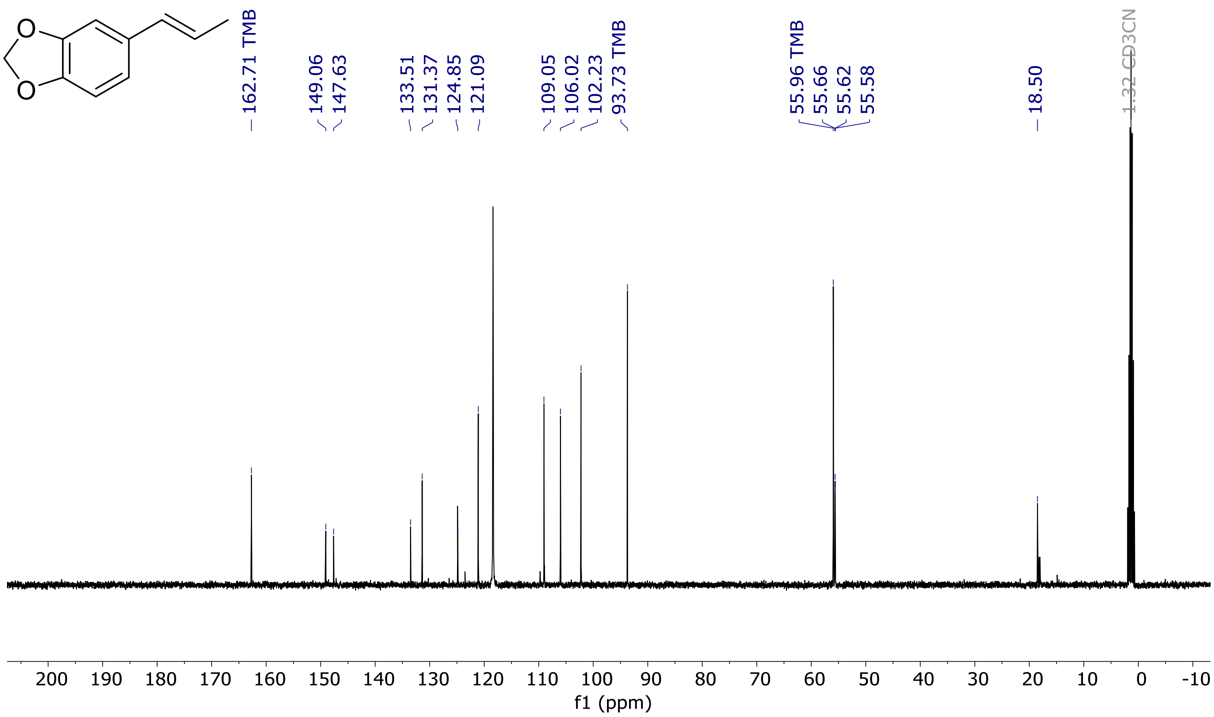

Figure S23. $^{13}C\{^{1}H\}$ NMR (101 MHz, CD_3CN) of (*E*)-Isosafrole, **2h**.

4. References

1. M. Mayer, A. Welther and A. Jacobi von Wangelin, *ChemCatChem*, 2011, **3**, 1567-1571.
2. H. Liu, M. Xu, C. Cai, J. Chen, Y. Gu and Y. Xia, *Org. Lett.*, 2020, **22**, 1193-1198.
3. N. Zhu, J. Zhao and H. Bao, *Chem. Sci.*, 2017, **8**, 2081-2085.
4. J.-H. Jeon, J.-Y. Yang, N. Chung and H.-S. Lee, *J. Agric. Food Chem.*, 2012, **60**, 12349-12354.
